# ATP6V_0_d2 controls *Leishmania* parasitophorous vacuole biogenesis via cholesterol homeostasis

**DOI:** 10.1371/journal.ppat.1007834

**Published:** 2019-06-14

**Authors:** Carina Carraro Pessoa, Luiza Campos Reis, Eduardo Milton Ramos-Sanchez, Cristina Mary Orikaza, Cristian Cortez, Erica Valadares de Castro Levatti, Ana Carolina Benites Badaró, Joyce Umbelino da Silva Yamamoto, Vânia D’Almeida, Hiro Goto, Renato Arruda Mortara, Fernando Real

**Affiliations:** 1 Departamento de Microbiologia, Imunologia e Parasitologia, Escola Paulista de Medicina, Universidade Federal de São Paulo, São Paulo, Brasil; 2 Laboratório de Soroepidemiologia e Imunobiologia, Instituto de Medicina Tropical, Universidade de São Paulo, São Paulo, Brasil; 3 Centro de Genómica y Bioinformática, Facultad de Ciencias, Universidad Mayor, Santiago de Chile, Chile; 4 Departamento de Bioquímica, Escola Paulista de Medicina, Universidade Federal de São Paulo, São Paulo, Brasil; 5 Departamento de Psicobiologia, Escola Paulista de Medicina, Universidade Federal de São Paulo, São Paulo, Brasil; 6 Departamento de Medicina Preventiva, Faculdade de Medicina, Universidade de São Paulo, São Paulo, Brasil; Institut national de la recherche scientifique, CANADA

## Abstract

V-ATPases are part of the membrane components of pathogen-containing vacuoles, although their function in intracellular infection remains elusive. In addition to organelle acidification, V-ATPases are alternatively implicated in membrane fusion and anti-inflammatory functions controlled by ATP6V_0_d2, the *d* subunit variant of the V-ATPase complex. Therefore, we evaluated the role of ATP6V_0_d2 in the biogenesis of pathogen-containing vacuoles using ATP6V_0_d2 knock-down macrophages infected with the protozoan parasite *Leishmania amazonensis*. These parasites survive within IFNγ/LPS-activated inflammatory macrophages, multiplying in large/fusogenic parasitophorous vacuoles (PVs) and inducing ATP6V_0_d2 upregulation. ATP6V_0_d2 knock-down decreased macrophage cholesterol levels and inhibited PV enlargement without interfering with parasite multiplication. However, parasites required ATP6V_0_d2 to resist the influx of oxidized low-density lipoprotein (ox-LDL)-derived cholesterol, which restored PV enlargement in ATP6V_0_d2 knock-down macrophages by replenishing macrophage cholesterol pools. Thus, we reveal parasite-mediated subversion of host V-ATPase function toward cholesterol retention, which is required for establishing an inflammation-resistant intracellular parasite niche.

## Author summary

V-ATPases control acidification and other processes at intracellular vesicles that bacteria and parasites exploit as compartments for replication and immune evasion. We report that the protozoan intracellular parasite *Leishmania amazonensis* resists inflammatory macrophage immune responses and upregulates an alternative isoform of subunit d of V-ATPase (ATP6V_0_d2). *Leishmania* are still sequestered within acidified parasitophorous vacuoles (PVs) in cells lacking ATP6V_0_d2, but these PVs do not enlarge in volume, a distinguishing feature of intracellular infection by these parasites. Cholesterol levels in ATP6V_0_d2-deficient cells are reduced and exogenous cholesterol repletion can restore vacuole size, leading to enhanced parasite killing. This study demonstrates the ATP6V_0_d2-mediated interplay of macrophage cholesterol retention and control of the biogenesis of large pathogen-containing vacuoles. The study provides grounds for the development of new therapeutic strategies for diseases caused by intracellular pathogens sheltered in host cell compartments.

## Introduction

Vacuolar H^+^-ATPases (V-ATPases) are membrane-associated ATP-dependent multimeric enzymes responsible for pumping protons from the cytosol into the lumen of intracellular organelles, thus controlling the acidification of lysosomes, endosomes, the trans-Golgi network and other intracellular vesicles [1, 2]. V-ATPases display two functionally distinct domains composed of several subunits: the cytosolic domain V_1_, composed of eight subunits (A, B, C, D, E, F, G and H) and that is implicated in ATP hydrolysis, and membranal domain V_0_, which is composed of subunits *a*, *d*, *e*, *c*, *c*’, and *c*” and is implicated in proton transport across the vesicle membrane [1].

Acidification of intracellular compartments is the canonical function of V-ATPases, which are largely implicated in diverse cellular processes, such as maturation and degradation of proteins, receptor-mediated endocytosis, receptor recycling and endocytic traffic [3, 4]. At the crossroads of innate immunity and endocytosis, V-ATPases are responsible for phagolysosome acidification in macrophages and other professional phagocytes, a key feature in the immune response against intracellular pathogens [5]. Maintenance of an acidic pH controlled by V-ATPases is required for the optimal activity of lysosomal digestive enzymes and production of hydrogen peroxide and other reactive oxygen species directly involved in pathogen killing [6].

Pathogens have nevertheless evolved strategies to evade phagolysosome acidification and killing, including targeting and subverting V-ATPase functions, thus improving their adaptation inside the hostile environment of host cells [7]. The pathogen-mediated subversion of V-ATPases may involve the interference of one or several subunits that compose the two functional domains, inhibiting proton pump activity or driving V-ATPases to target different organelles. The bacterial pathogens *Legionella pneumophila* and *Mycobacterium tuberculosis*, for instance, have the ability to secrete virulence factors that directly target the H*-*subunit of the V_1_ domain of host cell V-ATPases, blocking the acidification of bacteria-containing vacuoles in which they multiply by V-ATPase exclusion [8–10]. Conversely, *Yersinia pseudotuberculosis* does not exclude V-ATPases from the bacteria-containing vacuole but decreases their activity during intracellular infection [11].

In addition to coupling with the V_1_ domain and its proton translocation canonical function, the V_0_ membrane domain interacts with Soluble NSF Attachment Protein Receptors (SNAREs), thus being implicated in membrane fusion and exocytosis [12, 13]. These noncanonical functions of V-ATPases can take place when V_0_ domains are dissociated from V_1_ and directed to different organelles or when V-ATPases are composed of alternative isoforms of some of their subunits [4, 14, 15], a feature that could be exploited by intracellular pathogens.

The *a* subunit from the V_0_ domain, for example, has four different isoforms, each one expressed in different specialized cell types and distinct organelles [16]. The *d* subunit, also from the V_0_ domain, is expressed either as a ubiquitous isoform d1, which is implicated in the regular proton pumping activity of V-ATPases, or as an alternative isoform d2 (ATP6V_0_d2), which is highly expressed in restricted tissues, such as bones, kidney and lungs [17], and specialized cell types, such as osteoclasts [18] and macrophages [19], where it acts as a membrane fusogen [20–22].

The isoform ATP6V_0_d2 is implicated in counteracting macrophage inflammatory responses [23, 24]; therefore, the pathogen-induced production of this subunit isoform may constitute a mechanism by which intracellular pathogens multiply in macrophages despite inflammatory stimuli. Accordingly, ATP6V_0_d2 is upregulated in macrophages upon *in vitro* intracellular infection with the protozoan parasite *Leishmania (Leishmania) amazonensis* [25]. *Leishmania* spp. are trypanosomatid parasites, which induce tegumentary or visceral leishmaniasis in humans and other animals, a major health problem in poor and developing countries [26]. They are dimorphic parasites found extracellularly in the midgut of insect vectors as flagellated and elongated promastigotes and intracellularly in mammalian host macrophages, neutrophils and dendritic cells as round-shaped amastigotes [27]. Species from the *L*. *mexicana* complex, such as *L*. *amazonensis*, *L*. *mexicana* and *L*. *pifanoi*, are known to multiply within large and fusogenic pathogen-containing vacuoles or parasitophorous vacuoles (PV) [28], which are acidic compartments displaying functional V-ATPases [29]. Compared to other species, they also display, at least *in vitro*, a remarkable resistance to parasite killing mechanisms mediated by interferon-γ (IFN-γ) and lipopolysaccharide (LPS) within macrophages or by direct treatment with reactive oxygen species (ROS) [30–32]. A causal relationship between large PV development and parasite resistance to inflammatory macrophages remains elusive especially *in vivo*.

Considering that ATP6V_0_d2 participates in both membrane fusion and anti-inflammatory processes, we evaluated the participation of this subunit isoform in the biogenesis of pathogen-containing vacuole formation. ATP6V_0_d2 participation in *L*. *amazonensis* resistance to inflammatory macrophages upon stimulation with IFN-γ/LPS or treatment with inflammatory, oxidized lipoproteins (ox-LDL) was also approached. Here, we demonstrate that ATP6V_0_d2 is upregulated by intracellular parasites as a countermeasure to macrophage inflammatory immune responses, controlling the volumetric expansion of the pathogen-containing vacuole by regulating macrophage intracellular cholesterol levels. ATP6V_0_d2 does not participate in parasite survival within inflammatory macrophages classically activated by IFN-γ/LPS. ATP6V_0_d2 is required, however, for parasite survival within macrophages that scavenge ox-LDL via parasite-mediated increased expression of LOX-1 and CD36 scavenger receptors.

## Results

### ATP6V_0_d2 knock-down does not impact phagolysosomal acidification

**Fig 1:**
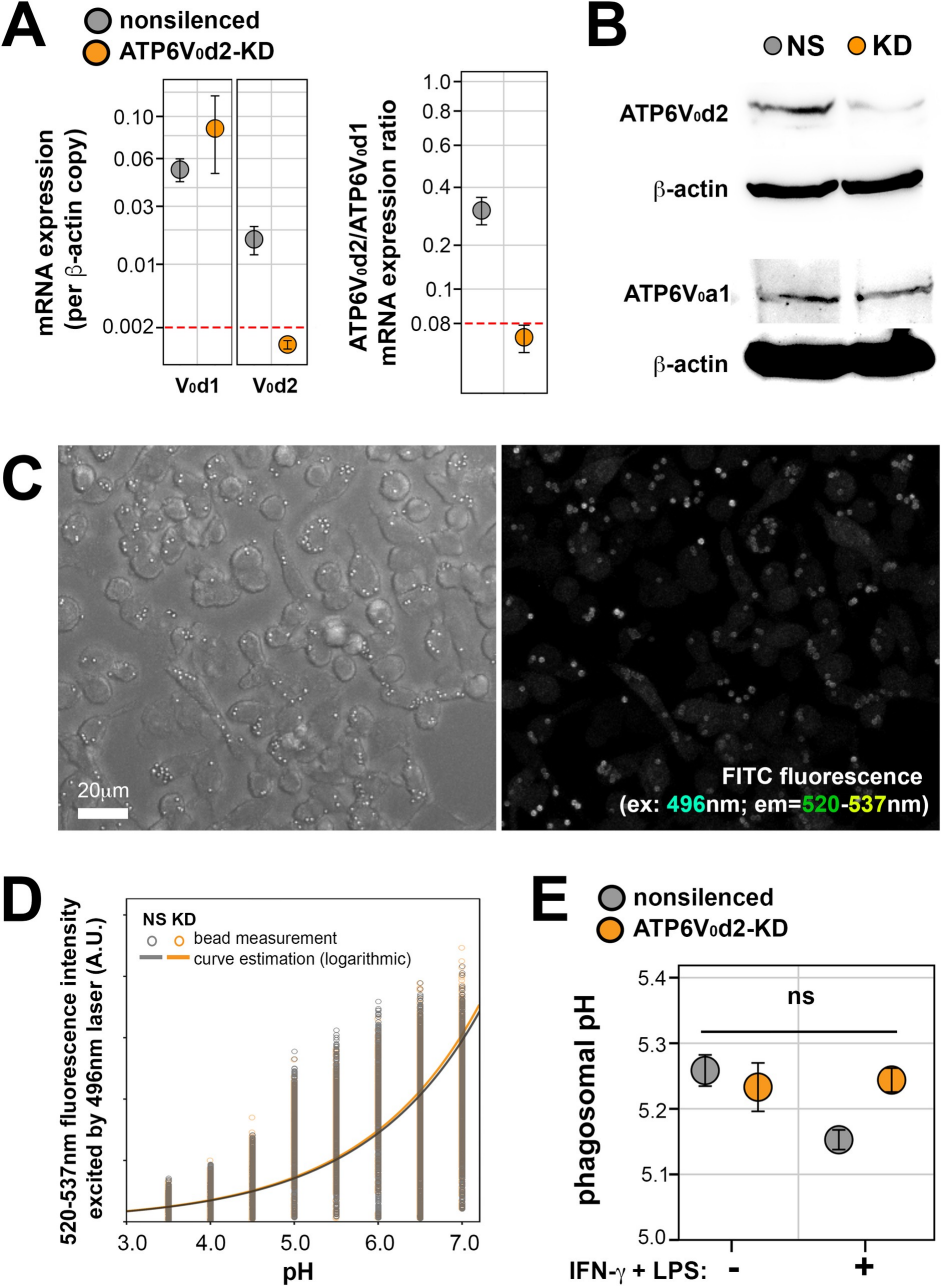
ATP6V_0_d2 knock-down does not impair phagolysosomal acidification. **A.** ATP6V_0_d2 and ATP6V_0_d1 mRNA relative expression in nonsilenced or ATP6V_0_d2-KD macrophages (left). ATP6V_0_d2 mRNA levels presented as a ratio between ATP6V_0_d2 and ATP6V_0_d1 expression. Dotted red line indicates level of knock-down. Results are representative of 5 independent experiments. **B.** Western blotting for ATP6V_0_d2 (38 kDa band) and ATP6V_0_a1 (116kDa) expression in nonsilenced (NS) or ATP6V_0_d2-KD macrophages (KD), confirming the specific silencing for d2 subunit and not for other components of the V_0_ complex. β-actin expression (42 kDa band) was assessed as loading control. **C-E.** Phagosomal pH evaluated in nonsilenced or ATP6V0d2-KD macrophages. **C.** Representative images of confocal microscopy showing FITC-coated latex beads that were engulfed by macrophages (differential interference contrast on the left and FITC green fluorescence on the right (FITC excitation at 496 nm; emission captured by 520–537 nm filter). Bar = 20 μm. **D.** FITC intensity (arbitrary units, AU, as obtained by microscope system) of each analyzed bead in macrophages cultivated at different pH (3.5–7.0) in nonsilenced (NS, gray) or ATP6V_0_d2-KD macrophages (KD, orange). A standard curve was generated from the mean values obtained at each pH condition and for each macrophage group (NS and KD). **E.** Average phagosomal pH of nonsilenced or ATP6V_0_d2-KD macrophages, activated or not by IFN-γ/LPS, estimated to be acidic between pH 5.1–5.3, in the conditions studied (ns = nonsignificant, p>0.05).

The subunit *d* (ATP6V_0_d) connects the two functionally distinct subunit V-ATPase complexes V_0_ and V_1_, which are responsible for the acidification of intracellular compartments. The subunit *d* from V-ATPase V_0_ complex occurs as two variants, ATP6V_0_d1 (ubiquitous) and ATP6V_0_d2, which expression is restricted to certain tissues and cells, expressed in parallel with ATP6V_0_d1 variant [17, 21]. V-ATPases will be thus composed of either d1 or d2 variant filling the space for the *d* subunit of V_0_ complex. To evaluate the role of isoform d2 in this canonical function of V-ATPases, we stably knocked-down ATP6V_0_d2 in RAW 264.7 macrophages (ATP6V_0_d2-KD) and evaluated phagolysosomal acidification using fluorescein (FITC)-tagged latex beads ingested by the phagocytes [33, 34]. We have stably and specifically knocked down the d2 variant (ATP6V_0_d2), not the ubiquitous ATP6V_0_d1 variant which predominates over ATP6V_0_d2 on nonsilenced control macrophages (Fig 1A). The expression of another V-ATPase subunit, ATP6V_0_a1, remains unaltered upon ATP6V_0_d2 knock-down (Fig 1B), demonstrating that this and likely all other subunits compose a functional V-ATPase in ATP6V_0_d2-KD macrophages. After phagosomal pH measurements using FITC-tagged beads internalized by nonsilenced and ATP6V_0_d2-KD macrophages (S1 Fig), we observed that, although ATP6V_0_d2 is efficiently knocked-down (Fig 1A and 1B), phagolysosomes containing FITC-tagged beads reach an acidic pH of approximately 5.2 in both nonsilenced and ATP6V_0_d2-KD macrophages, activated or not by IFN-γ/LPS treatment (Fig 1C–1E). Thus, the knock-down of ATP6V_0_d2 does not interfere in V-ATPase canonical function of phagolysosomal acidification as corroborated by others using different methods [21, 24].

Despite demonstrating that ATP6V_0_d2 does not participate in the V-ATPase canonical function of phagolysosome acidification, ATP6V_0_d2-KD macrophages display impaired lysosomal functions as assessed by analysis of the activity of some lysosomal enzymes. Cathepsin D (CTSD), one of the most well-studied lysosomal enzymes whose activity is a direct indicator of lysosomal functions [35, 36], was more abundantly associated with lysosome-associated membrane protein 1 (LAMP-1)-positive compartments as assessed by fluorescence colocalization analysis (S2A Fig), although cleaved, “mature” functional forms of CTSD were absent in ATP6V_0_d2-KD (S2B Fig). The activity of enzymes involved in lysosomal storage diseases that could indicate lysosome impairment was also evaluated: lysosomal acid lipase (LAL), implicated in Wolman and cholesteryl ester storage diseases, displayed the same activity in both nonsilenced and ATP6V_0_d2-KD macrophages; activity of α-galactosidase (α-Gal), implicated in Fabry Disease, was increased in ATP6V_0_d2-KD macrophages, while β-glucocerebrosidase (GCase) activity, whose activity deficiency is observed in Gaucher Disease, was decreased compared to nonsilenced macrophages (S2C Fig). All tested enzymes are acid hydrolases only active at acidic pH; considering that LAL activity does not depend on ATP6V_0_d2, we excluded an impairment of lysosome acidification in the lysosome dysfunction displayed by ATP6V_0_d2-KD macrophages. Therefore, ATP6V_0_d2 does not participate in the canonical V-ATPase function of phagolysosome acidification, instead exerting a pH-independent regulation of lysosomal enzymatic functions.

### Inhibition of ATP6V_0_d2 expression by IFN-γ/LPS stimulation is partially reversed by intracellular infection

**Fig 2:**
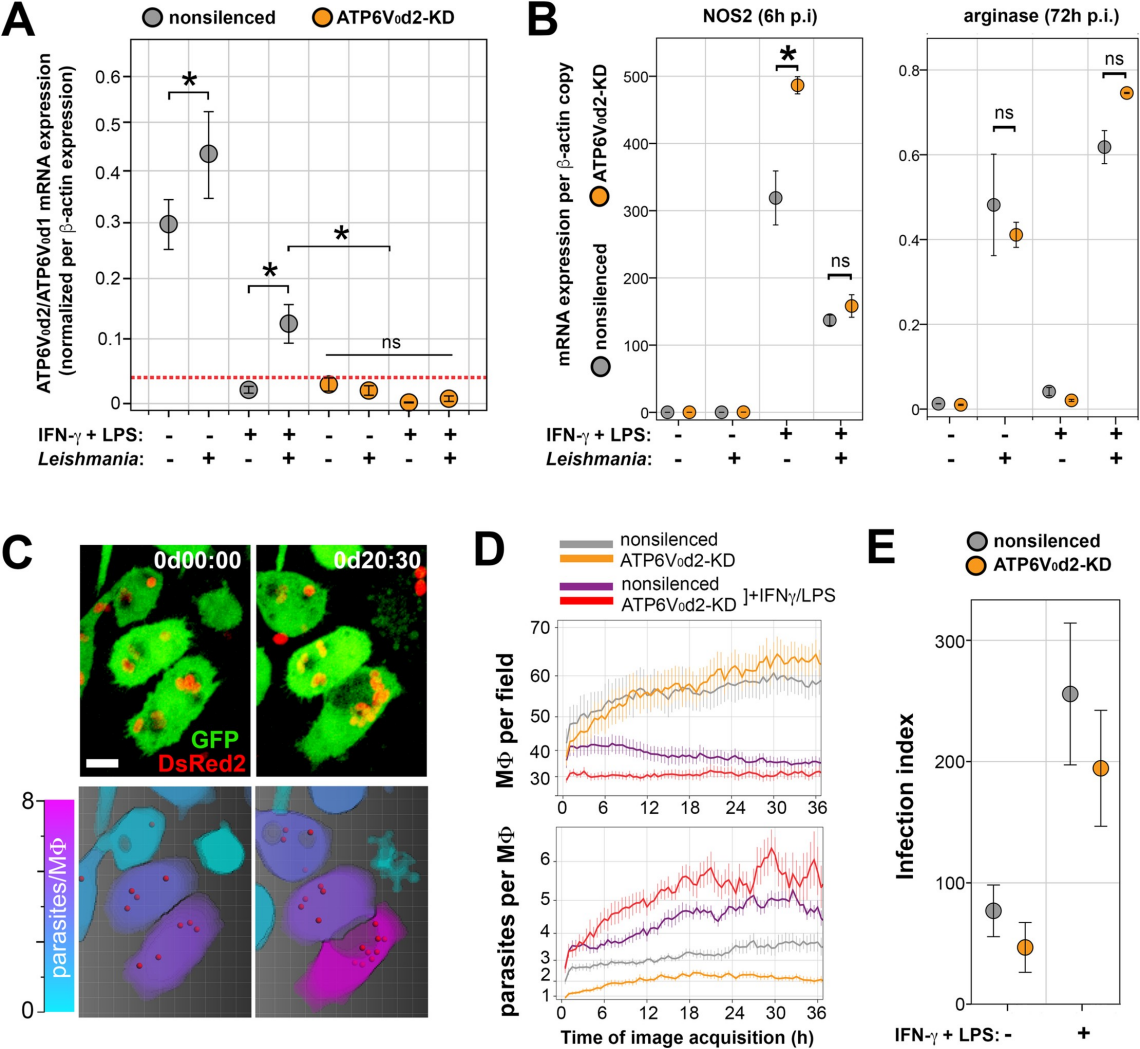
ATP6V_0_d2 upregulated by intracellular parasites does not participate in parasite resistance to IFN-γ/LPS-activated macrophages. **A.** ATP6V_0_d2 mRNA expression relative to expression of isoform d1 (ATP6V_0_d1) in nonsilenced or ATP6V_0_d2-KD macrophages activated or not with IFN-γ/LPS and infected or not by *L*. *amazonensis* for 48 hours. The results are representative of 5 independent experiments. *p< 0.05; ns = nonsignificant. **B.** Expression of NOS2 (left panel) and arginase (right panel) mRNA after 6 and 72 hours of *L*. *amazonensis* infection, respectively, in nonsilenced and ATP6V_0_d2-KD macrophages activated or not with IFN-γ/LPS. NOS2 and arginase mRNA expression was calculated relative to β-actin mRNA expression. The asterisks indicate statistical significance (p<0.05) between nonsilenced and ATP6V_0_d2-KD measurements. ns = nonsignificant. The results are representative of 2 independent experiments. **C-D.** Dynamic quantification of parasite numbers in macrophage cultures recorded by live imaging using image segmentation and automatic counting algorithms. In C, upper panel, images of GFP-expressing macrophages (green) merged with images of DsRed2-expressing parasites (red) at the start (0d00:00) and after 20 hours and 30 minutes (0d:20:30) of image acquisition. In the lower panel, the results of image segmentation processing, which identifies parasites (red spots) and macrophages using a color scale ranging from cyan (noninfected macrophage) to magenta (macrophage sheltering >8 parasites). Bar = 10 μm. In D, the number of macrophages per recorded field (upper graph) and the number of parasites per macrophage (lower graph) were assessed throughout 36-hour live imaging recordings of infected nonsilenced or ATP6V_0_d2-KD macrophages activated or not with IFN-γ/LPS. The data are represented as the means and standard errors of 8 different microscopic fields per condition. The results are representative of 2 independent experiments. **E.** Infection indexes obtained from nonsilenced or ATP6V_0_d2-KD macrophages activated or not with IFN-γ/LPS and infected with *L*. *amazonensis* for 72 hours. The results are representative of 3 independent experiments.

To evaluate the participation of ATP6V_0_d2 in the innate immune response of macrophages, we assessed the expression of ATP6V_0_d2 mRNA transcripts (relative to expression of its alternative ubiquitous isoform ATP6V_0_d1), in nonsilenced and ATP6V_0_d2-KD macrophages (Fig 2A). Macrophages were activated or not by IFN-γ/LPS treatment and cultured with or without the intracellular parasite *L*. *amazonensis* (S3A Fig). In nonsilenced macrophages, expression of ATP6V_0_d2 was upregulated upon *Leishmania* infection. We reproduced the remarkable decrease of ATP6V_0_d2 expression upon classical activation with IFN-γ/LPS as demonstrated by others [24], to levels comparable to those obtained in ATP6V_0_d2-KD macrophages. ATP6V_0_d2 expression is partially recovered by *Leishmania* intracellular infection, suggesting that *Leishmania* stimulates the expression of ATP6V_0_d2 as a countermeasure to the macrophage immune response.

However, ATP6V_0_d2 is not directly implicated in the macrophage responses related to parasite intracellular multiplication, namely: i) production of nitric oxide (NO) inferred by expression of the inducible isoform of nitric oxide synthase (iNOS, NOS2), the main effector of innate immunity against intracellular pathogens [37]; and ii) expression of arginase, which is involved in polyamine synthesis and is exploited by pathogens to establish intracellular infection [38]. NOS2 expression was increased upon IFN-γ/LPS treatment in ATP6V_0_d2-KD as compared with nonsilenced macrophages, indicating that ATP6V_0_d2 buffers this activation pathway in non-infected macrophages (Fig 2B, first graph). In infected macrophages, however, NOS2 expression was equally decreased upon IFN-γ/LPS treatment in nonsilenced and ATP6V_0_d2-KD macrophages harboring *Leishmania*, indicating that other host factors induced by the parasite, such as arginase, are more determinant in downregulating iNOS expression. Since arginase expression was increased in macrophages hosting the parasite independently of ATP6V_0_d2 knock-down or macrophage activation with IFN-γ/LPS (Fig 2B, second graph), the previous data showing decreased NOS2 expression upon IFN-γ/LPS treatment may be related to this increased arginase expression due to the presence of *Leishmania*.

### ATP6V_0_d2 controls the biogenesis of large *L*. *amazonensis* parasitophorous vacuoles without disturbing parasite multiplication

Multiplication of intracellular *Leishmania* was assessed by quantitative live imaging and microscopic counting (Fig 2C–2E). Cultures of macrophages infected with *Leishmania* were recorded by live imaging for 36 hours, and the numbers of macrophages per microscopic field and parasites per macrophage were quantified by image segmentation (Fig 2C). Independently of ATP6V_0_d2 knock-down, activation with IFN-γ/LPS inhibited RAW 264.7 cell proliferation (Fig 2D, upper graph) but increased *Leishmania* intracellular multiplication (Fig 2D, lower graph), as demonstrated by others upon IFN-γ-only treatment [30]. At the end of 72 hours after administration of parasites to macrophage cultures, samples were fixed, and the numbers of macrophages and parasites hosted per macrophage were converted into an infection index, which revealed that activation with IFN-γ/LPS increased parasite multiplication independently of ATP6V_0_d2 (Fig 2E).

**Fig 3:**
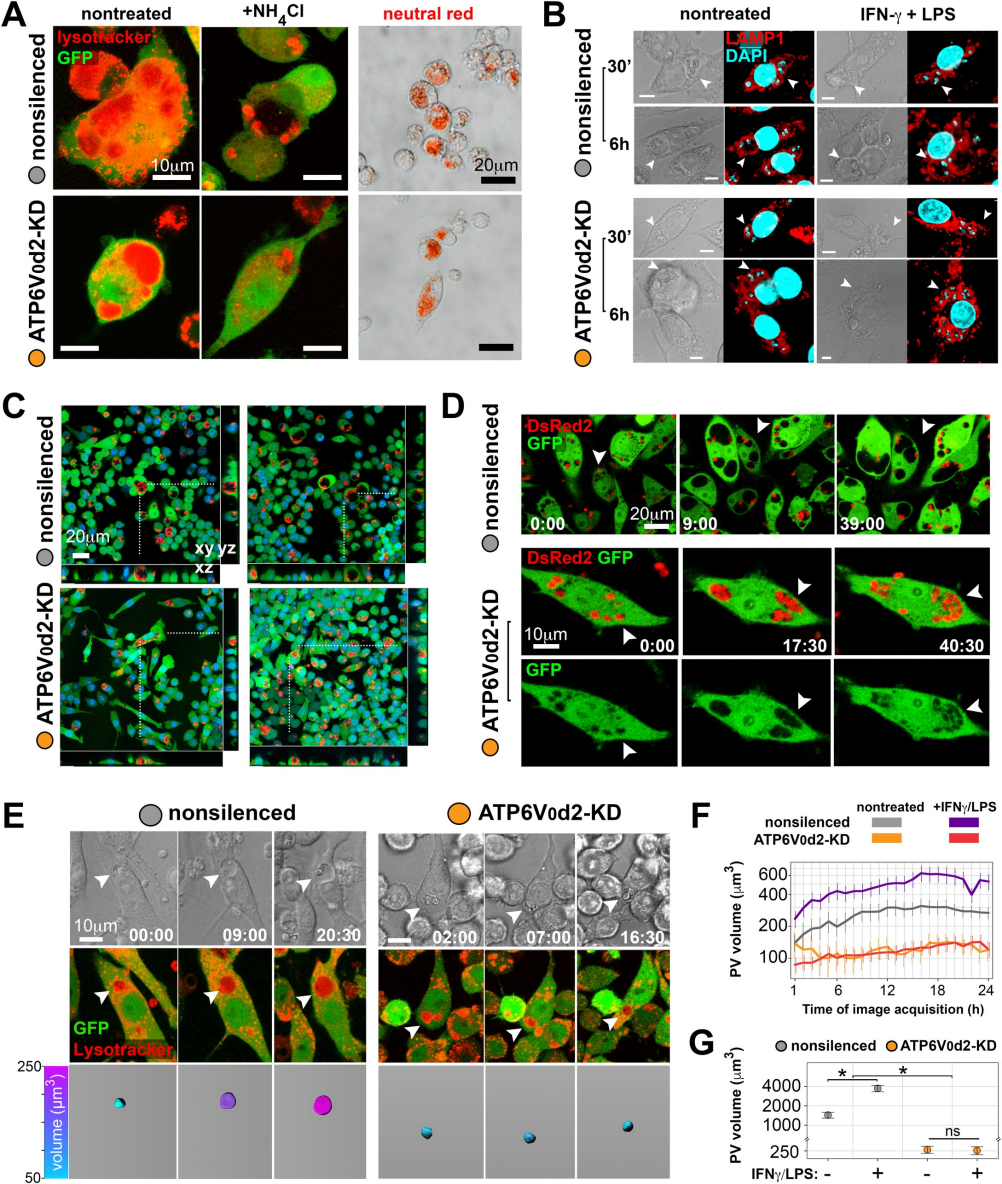
ATP6V_0_d2 controls the volumetric expansion of *L*. *amazonensis* parasitophorous vacuoles without interfering with their acidification or acquisition of lysosome membrane proteins. **A.** Confocal and phase contrast microscopy images showing nonsilenced or ATP6V_0_d2-KD macrophages that display acidified *L*. *amazonensis* PVs as assessed by a fluorescent lysosomotropic probe (first 2 columns, macrophages in green, lysosomotropic probe in red). Specificity of the probe for acidic compartments was confirmed by cultivating macrophages in the presence of the probe and ammonium chloride (NH_4_Cl, second column). Bar = 10 μm. Phagolysosomal acidification was confirmed by another lysosomotropic probe, Neutral Red, in both macrophage groups (third column). Bar = 20 μm. **B.** Immunofluorescence images showing LAMP-1 staining (red) in the membrane of *L*. *amazonensis* PVs (arrowheads) formed in nonsilenced or ATP6V_0_d2-KD macrophages activated or not with IFN-γ/LPS and hosting parasites for 30 minutes or 6 hours. Images show DIC and immunofluorescence for each analyzed group. Nuclei stained with DAPI. Bar = 5 μm. **C.** Two representative live imaging microscopic fields presenting the population of nonsilenced or ATP6V_0_d2-KD macrophages (green) infected by *L*. *amazonensis* (red) and their differences in PV dimensions as assessed by three-dimensional projections in *xy*, *xz* and *yz* coordinates. Dotted lines indicate the macrophages projected in three-dimensions. Images show nucleus staining by Hoechst live cell nuclear dye. Bar = 20 μm. **D.** Live imaging of IFN-γ/LPS-activated nonsilenced or ATP6V_0_d2-KD macrophages (green) hosting *L*. *amazonensis* (red). Arrowheads indicate PV volumetric expansion in nonsilenced macrophages or PV fission in macrophages silenced for ATP6V_0_d2. Time of image acquisition is expressed as hours:minutes (h:mm). Bars = 20 and 10 μm. **E.** Dynamic measurement of PV volumetric expansion from live imaging of infected nonsilenced or ATP6V_0_d2-KD macrophages (first row, differential interference contrast images), applying image segmentation on macrophages (green) and lysosomotropic probe (red) fluorescent channels (merged in the second row). The images represent the same infected macrophages recorded at different time points (presented as hours:minutes) of image acquisition. The lysosomotropic probe fluorescence retained in the PV allowed for reconstruction of the compartments as an isosurface from which volumetric information was assessed (third row, probe-positive detected PV represented in a colorimetric scale ranging from 50 μm^3^ in cyan to 250 μm^3^ in magenta). Bar = 10 μm. **F.** Dynamic tracking of PV volumetric expansion applied to infected nonsilenced or ATP6V_0_d2-KD macrophages, activated or not with IFN-γ/LPS, throughout a 24-hour live imaging acquisition. Parasites hosted by ATP6V_0_d2-KD macrophages are sheltered within smaller PVs with restrained PV volumetric expansion. The data are represented as the means and standard errors of 10 different microscopic fields per condition. The results are representative of 3 independent experiments. **G.** PV volumetric measures (n = ~50 vacuoles) of nonsilenced or ATP6V_0_d2-KD macrophages, activated or not with IFN-γ/LPS, in samples fixed after 48 hours of infection. The asterisks indicate statistical significance (p<0.05). ns = nonsignificant. The results are representative of 3 independent experiments.

Next, we evaluated *L*. *amazonensis* PV features, such as acidification and PV volumetric enlargement [28], in nonsilenced and ATP6V_0_d2-KD macrophages. Intracellular parasites are sequestered within acidified PVs independently of ATP6V_0_d2, as assessed by lysosomotropic probes retained in acidic compartments (Fig 3A). Complete abrogation of probe fluorescence of the *L*. *amazonensis* PV in macrophages treated with the alkalinizer agent ammonium chloride (NH_4_Cl) functionally confirmed the acidified content of PVs formed independently of ATP6V_0_d2. In addition, the trafficking of LAMP-1 to the *L*. *amazonensis* PV membrane, a distinguishing feature of lysosomes, phagolysosomes and *Leishmania* PVs [28], was not altered by ATP6V_0_d2 knock-down in control or IFN-γ/LPS-activated macrophages (Fig 3B). In addition, the frequency of *L*. *amazonensis* PVs displaying the late endosomal SNARE VAMP8 in their membranes is not altered by ATP6V_0_d2 knock-down (S4D Fig). Concerning PV morphology, however, *L*. *amazonensis* PV developed in ATP6V_0_d2-KD macrophages did not enlarge in size as compared with nonsilenced macrophages according to three-dimensional projections of images obtained from infected samples (Fig 3C and S4A Fig).

To further investigate this impairment in PV enlargement, ATP6V_0_d2-KD macrophages hosting *L*. *amazonensis* PV were dynamically tracked by live imaging (Fig 3D, S1 Movie). The parasite developed enlarging PVs in nonsilenced macrophages (Fig 3D, arrowheads, upper row); this was in contrast to ATP6V_0_d2-KD macrophages, in which PV dimensions are smaller and often fit parasite size, promoting PV fissions as the parasite multiplies (Fig 3D, arrowheads, lower row). Using fluorescent lysosomal probes and image segmentation analysis [28], we dynamically assessed PV volumetric enlargement in parasite-infected macrophages activated or not with IFN-γ/LPS, demonstrating that *L*. *amazonensis* PV enlargement depends on ATP6V_0_d2 (Fig 3E–3G). On average, infected nonsilenced and ATP6V_0_d2-KD macrophages do not differ in or change their cell sphericity over the course of 36 hours of multidimensional (S4B Fig) and, in contrast to PV area measurements, PV volumetric assessment is nevertheless not influenced by cell sphericity effects (S4B and S4C Fig). These results demonstrate the participation of ATP6V_0_d2 in controlling *L*. *amazonensis* PV volumetric expansion.

### ATP6V_0_d2 regulates macrophage cholesterol levels and builds up pathogen-containing vacuoles protective from ox-LDL-derived cholesterol accumulation

**Fig 4:**
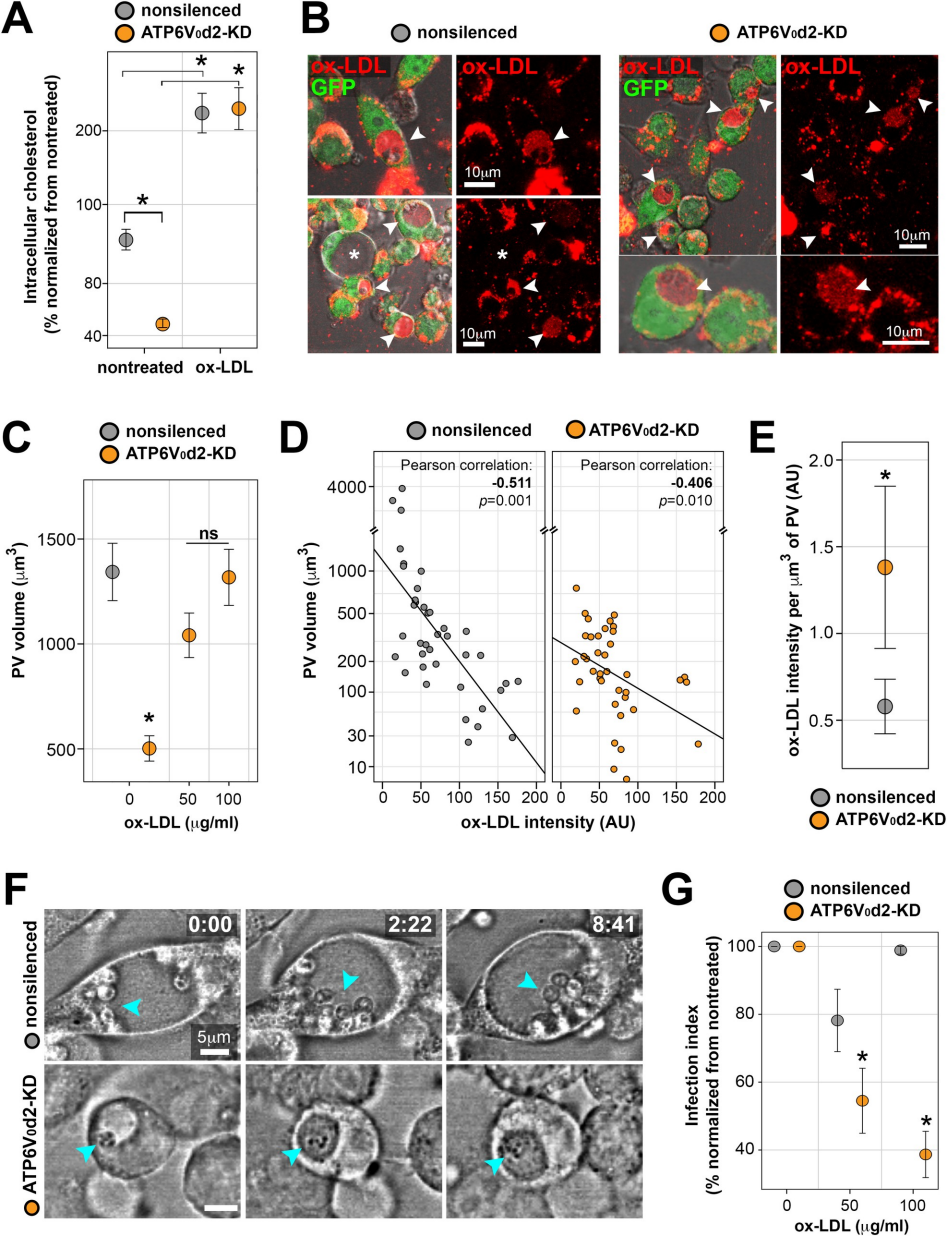
Ox-LDL-mediated repletion of ATP6V_0_d2-KD cholesterol levels restores PV volumes and impacts parasite multiplication. A. Intracellular cholesterol levels displayed by nonsilenced or ATP6V_0_d2-KD macrophages treated or not with 50 μg/ml of ox-LDL for 48 hours, showing that ox-LDL replenishes the ~40% lower cholesterol amount of ATP6V_0_d2-KD to levels comparable to nonsilenced macrophages. The data were normalized by the maximum value obtained in nontreated, nonsilenced macrophage. The asterisks indicate statistical significance (p<0.05), and the results are representative of 4 independent experiments. B. Confocal images of live infected nonsilenced or ATP6V_0_d2-KD macrophages (green) treated with fluorescent ox-LDL (Dil-ox-LDL). Arrowheads indicate PVs that accumulated ox-LDL and asterisks indicate a representative case in which ox-LDL are not retained in larger PVs. Bar = 10 μm. C. PV volumetric measurements (n = ~50 vacuoles) of ATP6V_0_d2-KD macrophages infected for 24 hours treated or not with 50 or 100 μg/ml of ox-LDL for the following 48 hours (comprising 72 hours of intracellular infection). The asterisks indicate statistical significance (p<0.05). ns = nonsignificant. The results are representative of 3 independent experiments. D. Correlation between PV volume and fluorescence intensities (in arbitrary units) of Dil-ox-LDL retained in PVs. PV isosurfaces were obtained from Dil-ox-LDL fluorescence signal, allowing for retrieving volumetric data. Larger PVs that do not accumulate ox-LDL were excluded from the correlation. Pearson’s correlation coefficients indicate statistically significant negative correlation between PV volumes and accumulation of ox-LDL in both nonsilenced and ATP6V_0_d2-KD macrophages. E. Amount of ox-LDL retained in PVs formed in nonsilenced and ATP6V_0_d2-KD macrophages expressed as Dil-ox-LDL fluorescence per μm^3^ of PV (* p<0.05). F. Time-lapse imaging in differential interference contrast of infected nonsilenced (upper row) or ATP6V_0_d2-KD macrophages (lower row) treated with 50 μg/ml ox-LDL. Image acquisition started 24 hours post-infection and 15 minutes after ox-LDL addition; time is represented as hours:minutes (h:mm). In the upper row, arrowheads indicate parasites multiplying in large PVs in nonsilenced macrophages in the presence of ox-LDL; in the lower row, arrowheads indicate parasite killing in PVs whose volume was restored in ox-LDL-treated ATP6V_0_d2-KD macrophages. Bar = 5 μm. G. Infection index calculated after 72 hours of infection (with or without 48 hours of cholesterol repletion with 50 or 100 μg/ml of ox-LDL) displayed by infected nonsilenced or ATP6V_0_d2-KD macrophages. Parasite multiplication impairments occurred specifically in ATP6V_0_d2-KD macrophages in a dose-dependent manner. The data were normalized per macrophage group (nonsilenced or ATP6V_0_d2-KD) by the maximum value obtained in nontreated macrophages. The asterisks indicate statistical significance (p<0.05) between nonsilenced and ATP6V_0_d2-KD indexes, and the results are representative of 5 independent experiments.

The biogenesis of large *L*. *amazonensis* PVs is accompanied by upregulation of host macrophage genes implicated in lipid metabolism, specifically cholesterol homeostasis [25], suggesting the participation of cholesterol in the intracellular establishment of this parasite. Therefore, we evaluated the intracellular levels of free cholesterol/cholesteryl esters in the studied macrophages, demonstrating that macrophages displayed a 40% decrease in cholesterol levels when ATP6V_0_d2 was knocked-down as detected by ELISA (Fig 4A, nontreated group) and confirmed by mass spectrometry (S5A Fig).

To functionally assess the participation of cholesterol in the ATP6V_0_d2-dependent biogenesis of *L*. *amazonensis* PVs, we envisioned a protocol for cholesterol repletion by adding oxidized low-density lipoprotein (ox-LDL) to macrophage cultures (S3B Fig), as performed previously [39–41]. Modified LDL, such as ox-LDL, is more efficiently taken up by macrophages through scavenger receptors and induces higher accumulation of intracellular cholesterol than native LDL [41, 42]. Among three different strategies to replenish macrophage intracellular cholesterol levels decreased in ATP6V_0_d2-KD–namely, treatment with methyl-β-cyclodextrin/cholesterol complexes [43], with LDL [41, 42] or with ox-LDL [39, 41]–ox-LDL was the most effective method to replenish intracellular cholesterol with less cytotoxicity in both nonsilenced and ATP6V_0_d2-KD macrophages (Fig 4A and S5B and S5C Fig).

Accumulation of ox-LDL-derived cholesterol in macrophages leads to the formation of foamy macrophages, which are full of lipid-laden vacuoles (lipid droplets) [44, 45] that could reconstitute *L*. *amazonensis* PV volumes in ATP6V_0_d2-KD macrophages. Accordingly, exogenous ox-LDL traffics into PVs independently of ATP6V_0_d2 (Fig 4B, arrowheads), and the ox-LDL-mediated intracellular cholesterol repletion in ATP6V_0_d2-KD macrophages hosting *L*. *amazonensis* increased the PV volume to dimensions comparable to those measured in nonsilenced macrophages (Fig 4C and S4C FIg). There is a negative correlation between PV size and the amount of ox-LDL accumulated within PVs, demonstrating that smaller PVs like those formed in ATP6V_0_d2-KD macrophages accumulate more ox-LDL (Fig 4D). Importantly, PVs formed in ATP6V_0_d2-KD macrophages—which recover their dimensions by ox-LDL treatment—retain more ox-LDL per μm^3^ as compared with PVs formed in nonsilenced macrophages (Fig 4E).

This ox-LDL-mediated PV dimensional recovery was accompanied by a decrease in the intracellular survival of *L*. *amazonensis* specifically within ATP6V_0_d2-KD macrophages, as assessed by comparing infection indexes under two different concentrations of ox-LDL (Fig 4F and 4G). Parasites hosted within PVs formed in ATP6V_0_d2-KD macrophages and enlarged after treatment with ox-LDL displayed aberrant morphology suggestive of parasite killing [46] in contrast to parasites multiplying in nonsilenced macrophages under the same ox-LDL treatment (Fig 4F and S2 Movie).

The ox-LDL-mediated PV size recovery observed in ATP6V_0_d2-KD macrophages is not related to differential expression of ATP6V_0_d subunit isoforms d1 and d2 (S6A Fig) or the differential expression of the lysosomal traffic regulator LYST/Beige (S6B Fig, right graph) involved in PV biogenesis [47]. In addition, the impaired intracellular establishment of *L*. *amazonensis* in ATP6V_0_d2-KD macrophages treated with ox-LDL was not due to increased production of reactive oxygen species [48] or inflammatory cytokines upon cellular uptake of ox-LDL [49] at the evaluated ox-LDL concentration (S6C Fig). Finally, the enzymatic activities of α-Gal and GCase lysosomal enzymes after ox-LDL-mediated cholesterol replenishment were assessed and do not explain neither the ox-LDL-mediated recovery of PV dimensions in ATP6V_0_d2-KD macrophages (compare infected macrophages treated or not with ox-LDL, S2D Fig).

### ATP6V_0_d2 controls PV volume in cooperation with ox-LDL scavenger receptor CD36

The cholesterol intracellular homeostasis in macrophages can be regarded as a balance between cholesterol biosynthesis that generates cholesterol precursors involved in the cholesterol biosynthetic pathways, cholesterol catabolism, and cholesterol uptake/efflux promoted by receptors for non-modified LDL and scavenger receptors for modified LDL [50]. To approach the participation of ATP6V_0_d2 in cholesterol homeostasis, we have evaluated the mRNA levels of scavenger receptors and of the sterol regulatory element-binding protein 2 (SREBP2) which controls expression of genes involved in cholesterol synthesis [51], in the context of ATP6V_0_d2 knock-down, infection with *Leishmania* and treatment with ox-LDL. The non-altered mRNA expression of SREBP2 observed in the conditions studied (S6B Fig, left graph) and the non-altered abundance of the cholesterol biosynthetic precursors squalene and lanosterol observed by mass spectrometry comparing nonsilenced and ATP6V_0_d2-KD macrophages (S5A Fig) indicate that ATP6V_0_d2 does not associate with cholesterol biosynthesis.

**Fig 5:**
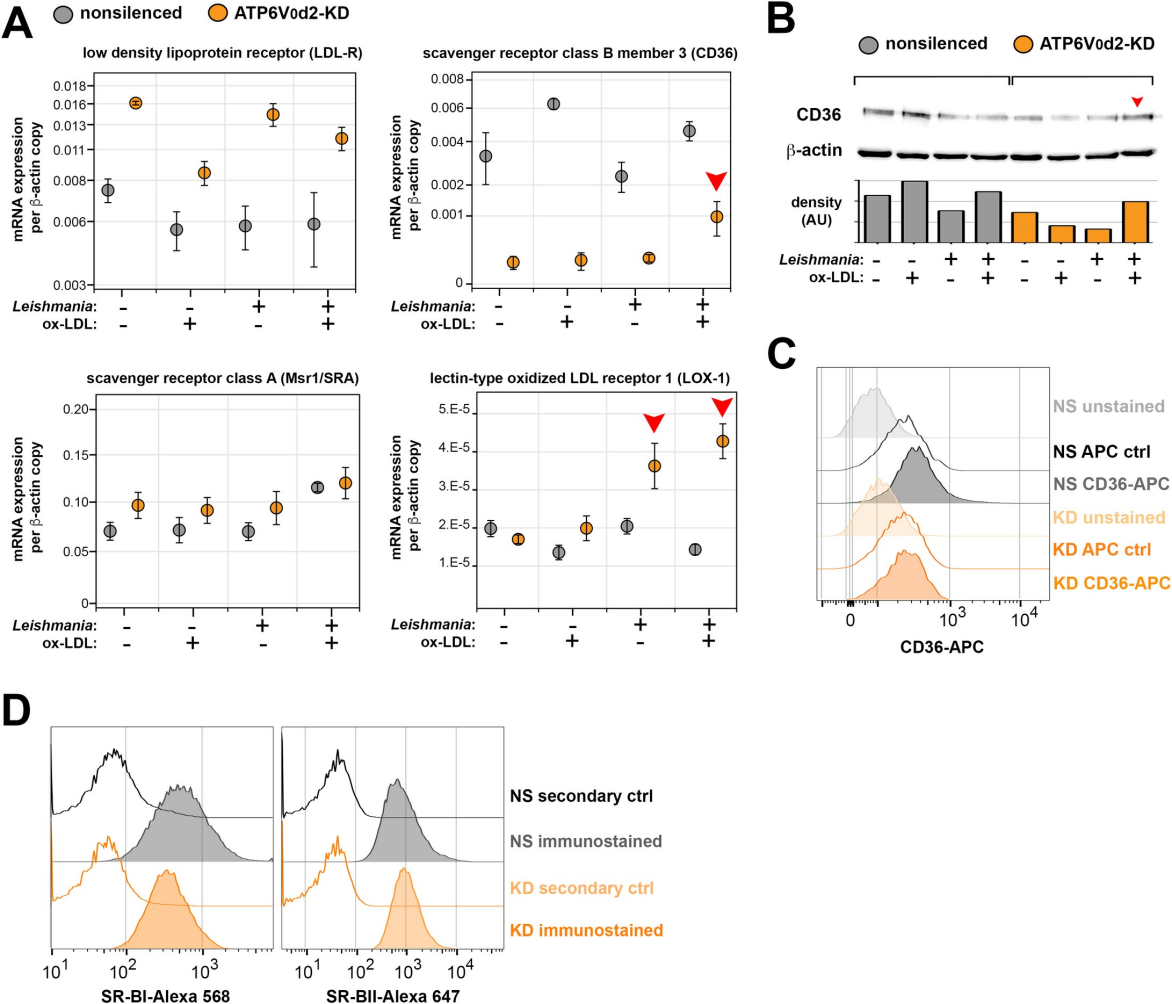
Parasite-mediated expression of macrophage scavenger receptor CD36 is associated with ox-LDL-mediated PV volume recovery in ATP6V_0_d2-KD. **A.** LDL-R, CD36, Msr1/SRA and LOX-1 mRNA expression in nonsilenced or ATP6V_0_d2-KD macrophages infected or not by *L*. *amazonensis* for 72 hours, treated or not with 50 μg/ml ox-LDL for 48 hours during intracellular infection. The results are representative of 2 independent experiments. Red arrowheads indicate parasite-mediated increase in mRNA levels of CD36 and LOX-1 scavenger receptors. **B.** Western blotting for CD36 protein expression (70-80kDa) in the studied conditions of infection and ox-LDL treatment. β-actin expression (42 kDa band) was assessed as loading control. CD36 expression detected by western blotting was further assessed by densitometric analysis of the protein bands corresponding to protein expression levels (graph on the lower panel, data expressed in arbitrary units). Red arrowhead indicates parasite-mediated increase in protein levels of CD36. **C.** CD36 membrane surface expression assessed by flow cytometry in nonsilenced (NS) or ATP6V_0_d2-KD macrophages (KD). Fluorescence intensities represented as FACS histograms; unstained controls and antibody isotype controls (APC ctrl) were employed to confirm CD36 antibody specificity. **D.** Membrane surface expression of scavenger receptors SR-BII and SR-BI in NS and KD macrophages assessed by flow cytometry. Fluorescence intensities represented as FACS histograms; fluorescence-coupled secondary antibodies without primary antibodies for the receptors (secondary ctrl) were employed to confirm antibody specificity.

An increased gene expression for LDL receptor (LDL-R) in ATP6V_0_d2-KD macrophages as compared with nonsilenced ones was observed independently of the conditions studied, with ox-LDL treatment decreasing the mRNA levels (Fig 5A, upper left graph). This is compatible with LDL-R stimulated expression upon lower intracellular cholesterol levels as displayed by ATP6V_0_d2-KD [52–54] and reinforces the role of ATP6V_0_d2 in the influx of cholesterol.

Considering the scavenger receptors for modified LDL, CD36 is decreased by ATP6V_0_d2 knock-down (Fig 5A upper right graph and 5B-C). RT-qPCR for CD36, covering the detection for all 5 isoforms of murine CD36, was the more efficient technique to detect these differences. The decrease of total (Fig 5B) and membrane surface (Fig 5C) CD36 levels was not so marked as the decrease observed in mRNA levels (Fig 5A). Recovery of PV dimensions by ox-LDL-mediated cholesterol replenishment in ATP6V_0_d2-KD occurs in parallel with increasing in CD36 gene expression specifically in infected ATP6V_0_d2-KD macrophages (Fig 5A and 5B, red arrowhead) in both mRNA and protein levels (Fig 5A upper right graph and 5B). Considering that the ox-LDL-mediated parasite killing occurs exclusively in ATP6V_0_d2-KD macrophages (parasites hosted by nonsilenced macrophages are resistant to ox-LDL intake) and that CD36 is known to control PV enlargement [55], we infer that CD36 participates in the recovery of PV dimensions upon ox-LDL uptake, what is detrimental to the parasite only in the absence of ATP6V_0_d2.

Other scavenger receptors implicated in ox-LDL intake display a non-altered expression in the conditions studied (Scavenger Receptor class A, Msr1/SRA, Fig 5A lower left graph) or display an increased expression specifically in infected ATP6V_0_d2-KD macrophages, although independent of ox-LDL treatment, such as the lectin-type oxidized LDL receptor 1, LOX-1 (Fig 5A, lower right graph). The membrane surface expression of scavenger receptors involved in cholesterol efflux, namely Scavenger receptor class B type 1 (SR-BI) and its alternative isoform SR-BII, was not altered by ATP6V_0_d2 knock-down (Fig 5D). Again, it reinforces the role of ATP6V_0_d2 in cholesterol intake in infected macrophages.

## Discussion

We report the participation of an alternative isoform of the V-ATPase subunit *d*, the isoform d2 (ATP6V_0_d2) in controlling the biogenesis of pathogen-containing vacuoles generated by *L*. *amazonensis* in macrophages. ATP6V_0_d2, whose expression is restricted to certain cell lineages, including macrophages, does not participate in phagolysosome acidification, indicating that the ubiquitous isoform d1 (ATP6V_0_d1) participates exclusively in the canonical function of this V-ATPase, while isoform d2 switches the V-ATPase toward noncanonical, acidification-independent functions, such as membrane fusion, regulation of lysosome enzymatic activities and downregulation of macrophage inflammatory burst [4, 21, 24, 56]. Therefore, the variant ATP6V_0_d1 is still expressed in ATP6V_0_d2 knock-down macrophages (ATP6V_0_d2-KD), capable of composing functional V-ATPases that acidify phagolysosomes and parasite-containing vacuoles. The preservation of phagolysosome acidification in the absence of the d2 variant demonstrated by us here and by others [21, 24] is a solid evidence that V-ATPases in ATP6V_0_d2-KD macrophages are functional and thus composed of all subunits required for their canonical functions.

ATP6V_0_d2 is involved in the function of important lysosomal enzymes, such as cathepsin D (CTSD), whose cleavage into mature forms depends on this V-ATPase subunit isoform. Inhibition of CTSD activity was demonstrated to either increase [57] or decrease [58] cholesterol intracellular levels depending on the studied models and a definitive participation of CTSD in cholesterol homeostasis remains to be established. Sphingolipid metabolism is also likely to be disturbed by ATP6V_0_d2 knock-down: β-glucocerebrosidase (GCase), whose activity is decreased in ATP6V_0_d2-KD macrophages and is responsible for breaking down glucosylceramide into ceramide [59], is also implicated in CTSD processing [60, 61], and α-galactosidase (α-Gal), whose activity is increased in ATP6V_0_d2-KD macrophages, participates in the production of glucosylceramide [62]. Hence, in addition to a 40% decrease in intracellular cholesterol levels, ATP6V_0_d2-KD macrophages could accumulate glucosylceramide (glucocerebroside) in detriment to ceramide and its incorporation into macrophage membranes. The data therefore indicate that ATP6V_0_d2 participates in lysosomal metabolic processes involved in the homeostasis of important membrane components, such as cholesterol and ceramide, which ultimately interfere in the biogenesis of pathogen-containing vacuoles in macrophages.

The regulation of lysosome function is coordinated by multiple factors, including proper assembly, trafficking and function of V-ATPases in the membrane of lysosomes and phagolysosomes. These lysosome-associated V-ATPase features could be controlled by ATP6V_0_d2 in macrophages reacting to pathogens and/or inflammatory stimuli. ATP6V_0_d2 is implicated in buffering inflammatory responses in macrophages, particularly upon TLR4 stimulation by LPS treatment [23]; however, the conclusion that this anti-inflammatory role of ATP6V_0_d2 is due to an ATP6V_0_d2-dependent vesicle acidification contrasts with our results and previous works showing that ATP6V_0_d2 depletion does not interfere in V-ATPase canonical functions such as ATP hydrolysis and H^+^ transport [21, 24] and that depletion of one particular subunit isoform does not interfere in V-ATPase-mediated phagosomal acidification, what would be compensated by expression with other variants (the case of subunit ATP6V_0_a3 [63]).

We demonstrated that ATP6V_0_d2 is upregulated by the parasite in IFN-γ/LPS-treated classically activated or M1-differentiated macrophages [64], e.g., macrophages that trigger an intra and extracellular inflammatory environment producing nitric oxide (NO) and reactive oxygen species (ROS), which is recognized as the most effective macrophage response against intracellular pathogens both *in vitro* and *in vivo* [30]. In contrast with *Leishmania major* parasites, which multiply in macrophages sheltered by tight-fitting pathogen-containing vacuoles and are sensitive to NO and ROS generated by classical macrophage activation, *L*. *amazonensis* and *L*. *mexicana* multiply within spacious and communal vacuoles and are resistant to M1 macrophage activation, that exerts cytostatic effects on intracellular *L*. *amazonensis* [28, 30, 31, 65, 66]. Conversely, our *in vitro* study demonstrated that macrophage stimulation with IFN-γ/LPS increased parasite multiplication independently of ATP6V_0_d2.

The persistence of this intracellular parasite despite inflammatory scenarios could be related to parasite-mediated counteraction of macrophage innate immune responses and microbicidal activities, e.g., by production of antioxidant enzymes to cope with oxidative burst [67] and establishment of a safe, customized intracellular niche where the parasite multiplies sheltered from ROS activity and antigen presentation [68, 69]. We reproduced the drastic downregulation of ATP6V_0_d2 expression upon LPS stimulation of macrophages as demonstrated by others [24], what is partially recovered by *Leishmania* infection. ATP6V_0_d2 is thus one of the several factors upregulated by the parasite in response to (or counteracting) the hostile environment of inflammatory macrophages. The ATP6V_0_d2-dependent volumetric expansion of pathogen-containing vacuoles may represent one additional countermeasure, possibly diluting phagolysosome hydrolases to concentrations innocuous to the parasite [70], thus favoring *L*. *amazonensis* multiplication. However, we observed that inhibition of PV volumetric enlargement by ATP6V_0_d2 knock-down did not interfere with parasite multiplication in either non-activated or IFN-γ/LPS-activated macrophages, suggesting that PV enlargement is not crucial for parasite intracellular multiplication and does not account for parasite persistence in NO-producing inflammatory macrophages, at least for a short 72-hour *in vitro* infection.

The ATP6V_0_d2-dependent PV expansion and parasite-mediated upregulation of ATP6V_0_d2 in IFN-γ/LPS-activated macrophages indicate that intracellular pathogens exploit ATP6V_0_d2 as a countermeasure to inflammatory scenarios. Although ATP6V_0_d2 does not participate in parasite resistance to the classical *in vitro* IFN-γ/LPS model of inflammatory macrophages, this V-ATPase subunit isoform was required for parasite survival in macrophages stimulated with ox-LDL, a potent inflammatory stimulus mainly studied in the context of atherosclerotic lesions but that has also been implicated in chronic psoriatic skin inflammation [71, 72].

Our results contrast with other mechanistic studies of *L*. *amazonensis* PV enlargement, which have established that interfering with the expression of host macrophage genes, such as the lysosomal traffic regulator LYST/Beige or some members of membrane fusion SNAREs machinery impact PV expansion and directly influence parasite multiplication [47, 73]. Parasite factors also account for this direct correlation between PV expansion and intracellular multiplication, as *L*. *mexicana* establishment in macrophages depends on Cysteine Peptidase B-mediated modulation of host cell membrane fusion machinery via the parasite GPI-anchored metalloprotease GP63 [73]. The observed PV impairments in these studies could be, however, the effect rather than the cause of parasite killing or inhibition of multiplication. We demonstrate that recruitment of late endosome-associated VAMP8 [74] to PVs and expression of LYST/Beige [47] are not associated with PV size impairments nor in the ox-LDL-mediated PV recovery observed in ATP6V_0_d2-KD macrophages. On the other hand, the main scavenger receptor for ox-LDL, CD36, was demonstrated to participate in the complex machinery that regulates PV biogenesis [55] and might be implicated in the ox-LDL-mediated PV dimensional recovery. The decreased CD36 expression in ATP6V_0_d2-KD macrophages together with increased LDL-R expression reinforce the central role of ATP6V_0_d2 gene on cholesterol intake and PV size. In addition, ATP6V_0_d2 knock-down, infection or ox-LDL treatment do not influence expression of SREBP2, which controls expression of genes involved in cholesterol synthesis [51]. Therefore, the ATP6V_0_d2-dependent PV biogenesis is unlikely to be related to cholesterol biosynthetic pathways but rather to cholesterol flux mechanisms. The similar expression of receptors involved in cholesterol efflux (SR-BI and SR-BII) in non-silenced and ATP6V_0_d2-KD macrophages, and the differences observed in the expression of receptors involved in cholesterol uptake strongly suggest that ATP6V_0_d2 participates in cholesterol influx.

**Fig 6:**
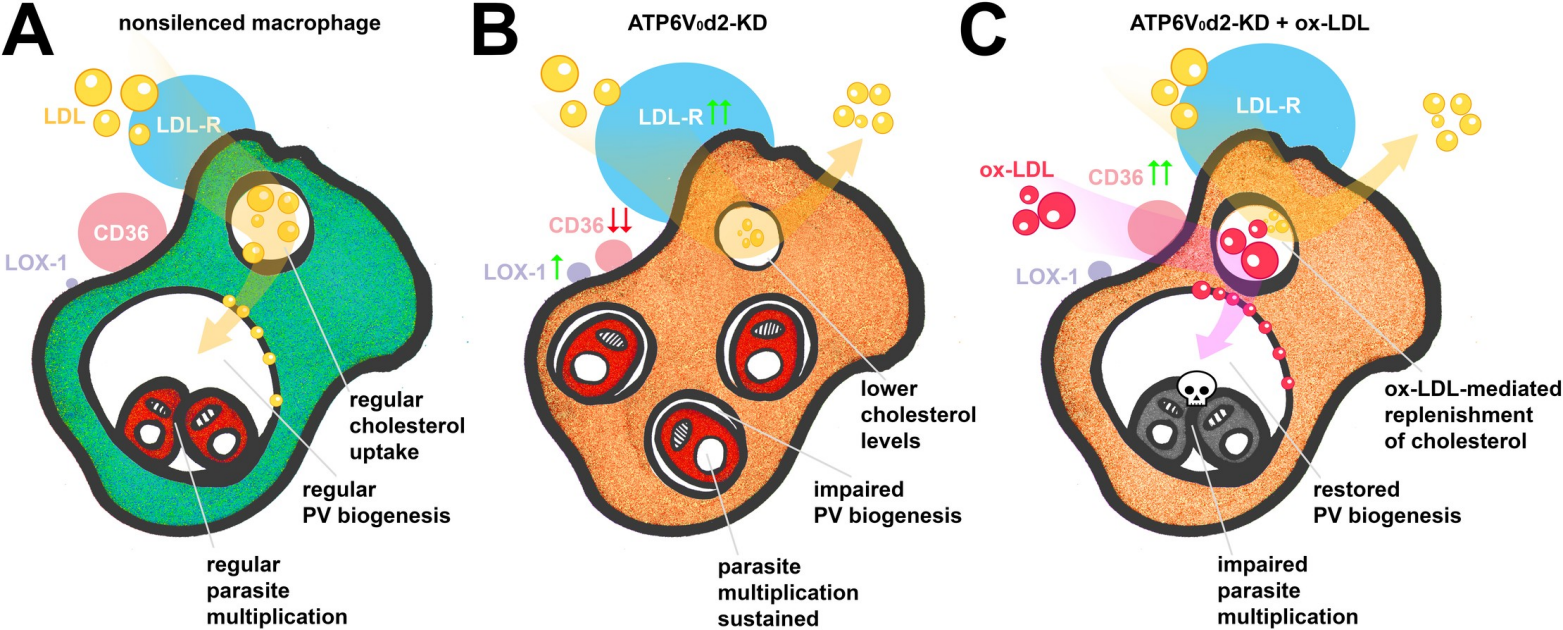
Mechanistic model proposed for the participation of V-ATPase subunit ATP6V_0_d isoform d2 in the macrophage intracellular cholesterol regulation and *L*. *amazonensis* PV biogenesis. **A.** The parasite upregulates the expression of d2 subunit (ATP6V_0_d2) but not the regular and ubiquitous d1 isoform, ATP6V_0_d1, indicating that infection is accompanied by additional functions of V-ATPases not necessarily related to acidification. Infection and PV biogenesis do not disturb the expression of cholesterol biosynthesis regulator SREBP2 nor the expression of scavenger receptors for nonmodified (LDL-R) or modified forms of LDL (Msr1/SRA, CD36, LOX-1) in nonsilenced macrophages. **B.** ATP6V_0_d2-KD macrophages presented 40% depletion in intracellular cholesterol levels that, although not interfering in parasite multiplication (which main sterol component is ergosterol), severely impaired *L*. *amazonensis* PV enlargement. CD36 expression is decreased during ATP6V_0_d2 knock-down and, with decreased levels of intracellular cholesterol, LDL-R expression is increased. LOX-1 and SRA expression suggests that modified LDL are able to be captured by infected cells despite ATP6V_0_d2 knock-down and CD36 decreased expression. **C.** When ATP6V_0_d2-KD macrophage intracellular cholesterol is replenished with exogenous oxidized Low-Density Lipoprotein (ox-LDL), a parasite-mediated restoration of CD36 expression is observed together with restored PV volumes. The ox-LDL source of cholesterol incorporated by ATP6V_0_d2-KD macrophages accumulated in PVs impaired parasite multiplication despite recovering PV dimensions.

While the precise molecular mechanisms controlling ox-LDL-mediated PV dimensional recovery and parasite killing working in cooperation with ATP6V_0_d2 remain to be elucidated, a model summarizing our results is presented in Fig 6. ATP6V_0_d2-KD macrophages displayed a 40% reduction in intracellular cholesterol levels, suggesting that the d2 subunit participates in cholesterol influx, which impacts the biogenesis of host cell membranes, including the formation of pathogen-containing vacuoles. Replenishment of ATP6V_0_d2-KD macrophage intracellular cholesterol levels with ox-LDL, modified LDL known to be more readily absorbed by macrophages compared with native LDL [42], partially reconstituted PV enlargement in parallel with parasite killing. The smaller the volume of PVs, the more ox-LDL is retained in these compartments, suggesting that as pathogen-containing vacuoles expand in volume, exogenous modified LDL internalized by macrophages are filtered out from or diluted within PVs. In this scenario, we speculate that, rather than induce an inflammatory cytokine microenvironment ultimately beneficial to the parasite [49, 75], the uptake of ox-LDL at the concentrations employed may induce the intracellular accumulation of oxygen radicals [76], oxidized phospholipids [77] and cholesterol crystals [71]. These compounds could access the parasites, and the potential anti-parasitic effects would be controlled by ATP6V_0_d2. The hypothesis that ATP6V_0_d2 induced by parasites during inflammation would, at the PV membrane level, restrict the access of LDL-derived components potentially toxic to intracellular parasites is in line with the demonstration that *Leishmania* does not have *de novo* cholesterol synthesis [78]. Furthermore, similar to other protozoan parasites, such as *Toxoplasma gondii*, *Trypanosoma cruzi* and *Cryptosporidium parvum* [79, 80], the parasite is able to salvage and incorporate host cell cholesterol through endocytosis of LDL [81, 82]. Importantly, *L*. *mexicana* is able to sequester host cell cholesterol directly from the large PV membrane built from exogenous LDL-derived components [83]. Therefore, PVs reconstituted in size by ox-LDL-mediated cholesterol influx in ATP6V_0_d2-KD macrophages (but not in nonsilenced macrophages) would be built up from ox-LDL-derived components potentially absorbed by the parasite, leading to parasite killing. ATP6V_0_d2 would participate in the selective features of *Leishmania* PV biogenesis, sparing the parasite from contacting and incorporating inflammation-derived toxic macrophage cargo.

This ATP6V_0_d2-mediated PV selectivity for ox-LDL-derived components could play an important role *in vivo*: *Leishmania* parasites developing large PVs are clinically associated with persistent diffuse granulomatous lesions in humans (diffuse cutaneous leishmaniasis), causing chronic damage to skin deep tissues despite only moderate inflammation in terms of NOS2 and IFN-γ expression compared to other disease manifestations [84, 85]. This context of persistent inflammation may favor the oxidative damage of proteins and lipids, resulting in oxidation and accumulation of modified LDL in tissues [48, 86], thus promoting an environment in which the ATP6V_0_d2-mediated selective PV biogenesis would account for *Leishmania* intracellular persistence. Therefore, ATP6V_0_d2 interference represents an unexplored therapeutic target for chronic diseases caused by inflammation-resistant intracellular pathogens.

Altogether, our results demonstrate that host macrophage V-ATPase functions can be subverted by the intracellular protozoan parasite *L*. *amazonensis*, thus establishing an intracellular niche in macrophages and allowing parasites to persist despite inflammatory environments.

## Material and methods

### Ethics statement

All experiments involving animal work were conducted under the guidelines approved by the Committee on the Ethics of Animal Experiments of the Institutional Animal Care and Use Committee at the Federal University of Sao Paulo (CEUA/UNIFESP n° 3398150715) in accordance with the Guide for the Care and Use of Laboratory Animals of the Brazilian National Council of Animal Experimentation (http://www.cobea.org.br/).

### Parasites

Wild-type MHOM/BR/1973/M2269 or DsRed2-transfected MPRO/BR/72/M1841 *L*. *(L*.*) amazonensis* amastigote parasites were derived from BALB/c mice footpad lesions and were maintained and obtained as described [87].

### Macrophage cultures

RAW 264.7 cells (macrophage-like cells, BALB/c origin and donated by Prof. Michel Rabinovitch, EPM-UNIFESP, São Paulo) were cultivated in RPMI medium supplemented with 10% fetal bovine serum (FBS), 100 U/ml penicillin, 100 μg/ml streptomycin (complete medium) and were incubated at 37°C in a humidified air atmosphere containing 5% CO_2_. Macrophages were stably silenced for ATP6V_0_d2 using GIPZ Lentiviral shRNAi transduction following the manufacturer’s instructions (Dharmacon, Inc.). Efficient transduction was monitored by GFP reporter gene expression. From three oligonucleotides tested (V2LMM_88448, V2LMM_194889 and V2LMM_88451), oligonucleotide V2LMM_88451 yielded >90% of ATP6V_0_d2 silencing, thus providing the preferred model of ATP6V_0_d2 knock-down (ATP6V_0_d2-KD) macrophages. Nonsilenced macrophage controls are macrophages stably expressing the GFP reporter gene and a nonsilencing shRNA which is processed by the endogenous RNAi pathway but its processed siRNA will not target any mRNA in the mammalian genome. The nonsilencing shRNA sequence is verified to contain no homology to known mammalian genes. Nonsilenced or ATP6V_0_d2-KD macrophages were cultivated in complete medium supplemented with 10 μg/ml puromycin until intracellular infection experiments. ATP6V_0_d2 efficient knock-down was confirmed up to 72 hours of intracellular infection or up to 96 hours after puromycin removal.

### Macrophage infection and infection index

*L*. *amazonensis* amastigotes were added to nonsilenced or ATP6V_0_d2-KD macrophages at a multiplicity of infection (MOI) of 20 parasites to 1 macrophage (20:1) for 6 hours of interaction at 34°C, 5% CO_2_. Macrophages were washed with phosphate-buffered saline (PBS) for the removal of non-internalized parasites, and complete medium was replenished without puromycin. Infected macrophages were maintained at 34°C, 5% CO_2_. The infection index was calculated 72 hours post-infection (p.i.) by multiplying the percentage of macrophages containing at least one parasite (% of infected macrophages) and the number of parasites per macrophage, as quantified after Giemsa counterstaining performed as described [88].

### Macrophage activation and ox-LDL treatment

Macrophages were treated with 20 ng/ml interferon-γ (IFN-γ) (R&D Systems, Inc.) and 1 μg/ml lipopolysaccharide (LPS) (Sigma-Aldrich Inc.) overnight and washed out before adding parasites to the macrophage cultures. Macrophages were infected for 24 hours prior to treatment with human high-oxidized low-density lipoprotein (ox-LDL, Kalen Biomedical, LLC, USA) diluted in complete medium for an additional 48 hours. Macrophage cultures were then washed with PBS and either incubated for 30 minutes with 200 nM Lysotracker Red DND-99 Invitrogen probe (for assessment of the volume of parasitophorous vacuoles) or proceeded to Giemsa staining for assessment of infection index. When indicated, infected macrophages were incubated with 50 μg/ml of fluorescent Dil-ox-LDL (Invitrogen L34358) for 48 hours.

### Laser scanning confocal microscopy

Images of paraformaldehyde 4%-fixed (PFA, Electron Microscopy Sciences) or live macrophage cultures infected with *L*. *amazonensis* were acquired with a Leica SP5 II Tandem Scanner System confocal unit (Leica Microsystems IR GmbH) coupled to a microincubator controlling the temperature and CO_2_ pressure conditions to 34°C, 5% CO_2_ (Tokai Hit Co., Japan). Fluorescence and Differential Interference Contrast (DIC) were acquired in the resonant scanning mode at 512 x 512 or 1024 x 1024 resolution using the 63× (HCX PL APO 63×/1.40–0.60 CS) or 100× (HCX PL APO 100×/1.44 CORR CS) immersion oil objectives, z-stacks between 0.5 to 0.8 μm and hybrid detectors enabled. During live imaging acquisitions, the lasers were adjusted to levels below 5% of laser power, and the duration of z-stacks was reduced to less than 30 seconds per recorded position to minimize phototoxicity. Images were processed by Imaris v.7.4.2 software (Bitplane AG, Andor Technology). Cells were stained for 15 minutes with Hoechst 33342 live cell nuclear dye (Thermo Fisher Scientific Inc.) as indicated.

### Dynamic quantification of parasites hosted by macrophages

Macrophages cultivated in ibiTreat-sterile tissue culture-treated HiQ4 multichamber dishes (ibidi GmbH) were infected with fluorescent *L*. *amazonensis* expressing DsRed2. These multichamber units allow for acquisition of four different experimental conditions at the same live imaging session, namely, infected nonsilenced or ATP6V_0_d2-KD macrophages activated or not with IFN-γ/LPS. Macrophage cultures were placed in the microincubator coupled to the confocal unit, and serial images of live, infected macrophages were acquired each 30 minutes during 36 hours in 8 microscopic fields per microchamber. A counting algorithm adapted from previous studies [28] was established using Imaris software as follows: i) isospots built based on parasite DsRed2 signals allowed for dynamic quantification of parasites per microscopic field during the acquisition period; ii) isosurfaces built based on macrophage GFP signals allowed for dynamic quantification of macrophages per microscopic field in the same acquisition period; iii) the ratio between these two variables per microscopic field provided the dynamic quantification of parasites per macrophages in infected cultures. The number of parasites in each analyzed macrophage was graphically represented by a color scale applied to each macrophage isosurface, ranging from cyan (no parasite) to magenta (>8 parasites per macrophage).

### Volumetric measurement of *L*. *amazonensis* parasitophorous vacuoles (PVs)

Macrophages cultivated in the HiQ4 multichamber dishes and infected with DsRed2-expressing *L*. *amazonensis* for 24 hours were incubated with 200 nM of Lysotracker Red DND-99 probe (Invitrogen) for a pulse of 30 min, washed and given fresh medium in the microincubator coupled to the confocal unit. The dynamic measurement of PV volumetric enlargement was performed as described [28], acquiring 10 microscopic fields per experimental condition. PV volumes in μm^3^ in each analyzed macrophage were graphically represented by a color scale applied to each PV isosurface, ranging from cyan (smaller) to magenta (larger PV). PV volume isosurfaces were also obtained from Dil-ox-LDL fluorescence for correlations between PV size and ox-LDL PV accumulation, using the same methodology. Similar to volume, cell sphericity is a measure obtained from three-dimensional image reconstructions assessed as described [87].

### Immunostaining for fluorescence microscopy and flow cytometry

Macrophages cultivated on 13 mm circular coverslips were fixed with 4% PFA in PBS and blocked for 30 minutes with 0.25% gelatin, 0.1% NaN_3_ and 0.1% saponin PBS solution prior to 1-hour incubation with primary antibodies, including 1:2 (v/v) rat anti-LAMP-1 (Developmental Studies Hybridoma Bank 1D4B) or 1:1000 (v/v) anti-cathepsin D (Abcam ab75852). Next samples were treated for 1 hour with a 1:100 (v/v) solution of anti-rat or anti-rabbit AlexaFluor-568 secondary antibodies (Invitrogen). Samples processed for confocal microscopy were treated for 15 min with 10 μM 4’,6-diamindino-2-fenilindol hydrochloride (DAPI) to stain macrophages and parasite nuclei. The coverslips were mounted with Dako Fluorescent Mounting Medium (Dako) before image acquisition under the confocal unit. Zymosan (Zymosan A Z-4250, Sigma-Aldrich Inc.) were administrated to macrophage cultures for 6 hours (50 particles per macrophage) for generation of 48-hours phagolysosomes used as positive control for VAMP8^+^ phagosomes, immunostained as described[68].

Samples processed for flow cytometry analysis were centrifuged 300*g* at 4°C for 5 minutes and incubated with BALB/c mouse serum for 1 hour to block Fc receptors in MACS buffer (PBS pH 7.2, 0.05% BSA, 2 mM EDTA). Then, cells were fixed by adding 400 μl of 1% PFA in 100 μl of MACS buffer for 30 minutes, washed and incubated with primary antibodies anti-CD36 (cat 552544 BD) 1:40 (v/v), anti-SR-BI (bs-1186R Bioss) 1:50 (v/v) or anti-SR-BII conjugated with AlexaFluor-647 (bs-7545R Bioss) 1:100 (v/v) in MACS buffer for 1 hour at 4°C. Fluorescence-coupled secondary antibodies were incubated for additional 1 hour at 4°C and include biotin anti-mouse IgA (cat 556978 BD) 1:500 (v/v) plus streptavidin-APC (cat 17-4317-82 eBioscience) 1:500 (v/v) (for CD36 antibody) and anti-rabbit AlexaFluor-568 1:100 (v/v) (for SR-BI antibodies). Then, cells were washed, centrifuged and resuspended in MACS buffer for analysis on LSR Fortessa cytometer (BD Biosciences). Unstained cells and cells treated with secondary antibodies alone were used as controls.

### Western blotting

Macrophage lysates were obtained by treating cultures with lysis buffer (Tris-HCl 50 mM pH 7.4, NaCl 150 mM, EDTA 1 mM, Triton X-100 1%) supplemented with a protease inhibitor cocktail (Halt Protease Inhibitor Cocktail, Thermo Fisher Scientific Inc.) at 4°C for 30 min and processed as described [88]. The membranes were blocked with TBS-Tween 0.1% buffer supplemented with 5% bovine serum albumin (BSA) for 1 hour. The primary antibodies rabbit anti-ATP6V_0_d2 (Sigma-Aldrich Inc. SAB2103220) 1:1000 (v/v), rabbit anti-ATP6V0a1 (Synaptic Systems cat 109 003) 1:1000 (v/v), rabbit anti-LAMP-1 (Cell Signaling 9091S) 1:1000 (v/v), mouse anti-CD36 (BD cat 552544) 1:1000 (v/v), mouse anti-β-actin (Cell Signaling 8H10D10 #3700) 1:5000 (v/v) and rabbit anti-cathepsin D (Abcam ab75852) 1:1000 (v/v) were incubated in TBS-Tween 0.1% supplemented with 5% bovine serum albumin overnight at 4°C. Anti-rabbit (A6154, Sigma-Aldrich Inc.) and anti-mouse (Sigma-Aldrich Inc. A4416) IgG peroxidase 1:8000 (v/v) secondary antibodies were incubated with 5% BSA in TBS-Tween 0.1% for 1 hour at room temperature. Biotin anti-mouse IgA (BD cat 556978) 1:8000 and Streptavidin-HRP (Southern Biotechnology Assoc. Inc cat 7100–05) 1:8000 (v/v) secondary antibodies were used to detect CD36 and incubated with 5% BSA in TBS-Tween 0.1% for 1 hour at room temperature. The membrane images were acquired using ECL Prime reagent (GE Healthcare Life Sciences) and analyzed on a UVITEC photodocumentator (Cleaver Scientific Ltd). Protein bands were quantified by densitometry using AlphaEaseFC software 3.2 beta version (Alpha Innotech Corporation, San Leandro, CA, USA), and the results are expressed in arbitrary units, which were calculated by integrating the intensity of each pixel over the spot area and normalizing to the gel background.

### Real time quantitative polymerase chain reaction (RT-qPCR)

Macrophage messenger RNA (mRNA) was obtained and processed for quantitative RT-PCR as described [89]. The following primers for mouse sequences were employed in the RT-PCR analysis: *Mus musculus* ATPase, H+ transporting, lysosomal V0 subunit D2 (Atp6v0d2)—GenBank (access number: NM_175406.3), Forward: 5'- TGT GTC CCA TTC TTG AGT TTG AGG -3' and Reverse: 5'- AGG GTC TCC CTG TCT TCT TTG CTT -3'; subunit d1 (NM_013477.3), Forward: 5’-ATT GGC CAG GAA GTT GCC ATA AT-3’ and Reverse: 5’-GTC GTT CTT CCC GGA GCT CTA TTT-3’; Arginase 1 (NM_007482.3) Forward: 5′-AGC ACT GAG GAA AGC TGG TC- 3′ and Reverse: 5′-CAG ACC GTG GGT TCT TCA CA-3′; Nos2 (NM_010927.4) Forward: 5′- AGA GCC ACA GTC CTC TTT GC- 3′ and Reverse: 5′- GCT CCT CTT CCA AGG TGC TT- 3′; Lysosomal trafficking regulator (NM_010748.2) Forward: 5´- GCC TGG ATG AAG AAT TTG ATC TGG-3´and Reward: 5´- ATT AGT CCG AGA ACG GGA ATG ACA-3´; Sterol regulatory element binding factor 2 (Srebf2) (NM_033218.1) Forward: 5´- ACC AAG CAT GGA GAG GTA GAC ACC-3´ and Reverse: 5´- GGA AGA CAG GAA AGA GAG GGG AAG-3´; CD36 molecule (NM_001159558.1) Forward: 5´- GGC TAA ATG AGA CTG GGA CCA TTG-3´ and Reverse: 5´- AAC ATC ACC ACT CCA ATC CCA AGT-3´; Low density lipoprotein receptor (LDLR) (NM_010700.3) Forward: 5´- AAC CTG AAG AAT GTG GTG GCT CTC-3´and Reverse: 5´- CAT CAG GGC GCT GTA GAT CTT TTT-3´; Lectin-like oxidized low-density lipoprotein receptor-1 (Lox-1) (NM_138648.2) Forward: 5’- TCT TTG GGT GGC CAG TTA CTA CAA -3’ and Reverse: 5’-GCC CCT GGT CTT AAA GAA TTG AAA-3’; Scavenger receptor class A (SRA) (NM_031195.2)Forward: 5’- CTA CAG CAA AGC AAC AGG AGG ACA– 3’ and Reverse: 5’–TGC GCT TGT TCT TCT TTC ACA GAC- 3’. For all experiments, β-actin and HPRT were used as the endogenous gene. β-actin (NM_007393.5) Forward: 5´-GCC TTC CTT CTT GGG TAT GGA ATC-3´ and Reverse: 5´-ACG GAT GTC AAC GTC ACA CTT CAT -3´; HPRT (NM_013556.2) Forward: 5´-TCA GTC AAC GGG GGA CAT AAA AGT-3´and Reverse: 5´- ACC ATT TTG GGG CTG TAC TGC TTA-3´.

Gene expression analysis is in accordance with the MIQE guidelines [90]. We present results using two endogenous genes, i.e. β-actin and HPRT, showing that the profile of the results is similar using both endogenous genes (S7D Fig). The efficiency of all the primers used is shown as values of slope, R^2^ and percentage of efficiency (S7A and S7B Fig). The parameter between the curves of target and endogenous genes of a standard curve is used to calculate the amplification efficiency of the reaction, according to the equation: E = [10^(-1 / slope)^– 1] x 100. The standard curve is obtained by linear regression of the Ct amplification (cycle threshold) value on the log of the initial cDNA amount. An angular coefficient of the standard curve of -3.32 indicates a reaction with 100% efficiency. Reactions are considered efficient when amplification efficiencies of the target and endogenous gene are very close, with a tolerance of ± 10% of variation [91]. The specificity of the qPCR reaction was demonstrated by the melt curves of each gene (S7C Fig). The data were presented as a relative quantification and were calculated using 2^−ΔΔCt^ [92].

### Assessment of phagolysosome acidification

To confirm acidification of *L*. *amazonensis* PVs, macrophages cultivated in HiQ4 multichamber dishes were infected for 24 h and then incubated for 20 minutes with 200 nM Lysotracker Red DND-99 or 100 μg/ml of Neutral Red dye before direct observation by confocal or bright-field microscopy, respectively. To test the specificity of the Lysotracker lysosomal probe for acidic pH, macrophages were treated with 10 mM ammonium chloride (NH_4_Cl) during probing.

To assess phagolysosomal pH, ATP6V_0_d2-KD or nonsilenced macrophages were cultivated in HiQ4 multichamber plates in the presence of FITC-coated latex beads (20 beads per macrophage) for 24 hours at 34°C, 5% CO_2_. Fluorescein fluorescence intensity decreases in direct correlation with acidic pH [33] and we have explored the differences in the excitation maximum of turboGFP (ex. max = 482 nm) and FITC (ex. max = 495 nm) to specifically detect fluorescence from FITC using Leica hybrid photodetectors (Leica HyD). When excited by a 496 nm laser (400 Hz frequency and 10% laser power), FITC is detected by Leica HyD 2.7 more efficiently then turboGFP using an emission range of 520–537 nm (S1A Fig), allowing us to adjust the voltage (gain) of photodetectors to threshold out turboGFP emission (S1B Fig). The raw acquired image of FITC beads are cleared from turboGFP fluorescence overlap (S1B Fig), and the fluorescence intensities per FITC-tagged bead are retrieved (in arbitrary units generated by Leica system, Fig 1C–1E). For each field, a *z* series of 18 images (steps) in resolution of 512 x 512 pixels and an average of 3 scans per line (line average) were established. FITC fluorescence intensity per bead was retrieved from bead isospots built using Imaris software as described [28]. This approach was applied to FITC-tagged beads internalized by GFP-expressing non-silenced and ATP6V_0_d2-KD macrophages incubated in complete medium adjusted to different pH ranging from 6.5–5 (buffered with 15 mM HEPES) to 4.5–3.0 (buffered with 30 mM citrate buffer) and in the presence of 10 μM of the ionophore nigericin (Sigma-Aldrich Inc.), which will rapidly equilibrate the pH within phagosomes with that of the extracellular medium [5]. A standard curve of pH measurement was then obtained using both non-silenced and ATP6V_0_d2-KD macrophages (Fig 1D), generating very similar functions positively correlating pH and the FITC fluorescence acquired that validate the method applied in this particular condition (i.e., FITC-tagged beads within GFP-expressing cells). The mean FITC fluorescence intensities retrieved in each experimental group were applied to the standard curve to obtain phagosomal pH.

### Assessment of lysosomal enzyme activity

α-galactosidase and β-glucocerebrosidase activities were determined as described [93, 94], with modifications. The determination of the activity of these enzymes is based on its action on the fluorogenic substrate 4-methylumbiliferiferone-D-galactopyranoside/4-methylumbiliferone-D-glucopyranoside (Sigma-Aldrich Inc.), resulting in release of the 4-methylumbiliferone molecule (4MU) and allowing for inference of the enzymatic activity in nmol per mg of protein per hour. Determination of the activity of the lysosomal acid lipase (LAL) enzyme in cells was performed as described [95], with modifications. For this, the fluorogenic substrate 4-methylumbiliferone palmitate (4MU palmitate, Santa Cruz Biotechnology) was used in the presence of an LAL activator, cardiolipin, and an inhibitor, Lalistat (Sigma-Aldrich Inc.), that allows quantification of the enzymatic activity in nmol per mg of protein per hour.

### Intracellular cholesterol replenishment and measurement

Replenishment of macrophage intracellular cholesterol levels was performed as previously described using methyl-β-cyclodextrin/cholesterol complexes [43], with LDL [41, 42] or ox-LDL [39, 41]. Methyl-β-cyclodextrin/cholesterol complexes were obtained by mixing 5 mM cholesterol (Sigma Aldrich C-8503) and 40 mM MβCD (Sigma Aldrich M-4555) in serum-free and non-antibiotic medium (macrophage-SFM 1X Gibco 12065–074). The solution was subjected to sonication for complete solubilization and incubated under shaking at 37°C overnight. Next, solution was filtered through a 0.45 μm filter and used in macrophage cultures. The concentrations of methyl-β-cyclodextrin/cholesterol complexes employed in the study refers to the 5mM cholesterol concentration used for composing the complexes. LDL was generously provided by Dr. Magnus Gidlund and Dr. Henrique Fonseca (University of São Paulo).

For intracellular cholesterol measurement, macrophages were lysed with lipid buffer (0.5 M potassium phosphate pH 7.4, 0.25 mM cholic acid and 0.5% Triton X-100) and sonicated in three high intensity cycles for 10 seconds [96], and cell lysates were then assessed for cholesterol levels by the Amplex Red Cholesterol Assay Kit (Thermo Fisher Scientific Inc.) according to the manufacturer's instructions. The results were normalized by the amount of protein obtained in lysates, as assessed by the Bradford method [97].

### Evaluation of cholesterol levels by atmospheric pressure chemical ionization

Total lipids were obtained from 2 x 10^7^ macrophages as described [98]. Purification of sterols was performed in a 10 x 2.5 cm silica gel 60 column (Merck Millipore). Samples were prepared using 10 μL of the sterol fraction (resuspended in 100 μL of methanol for each 10^7^ cells) in 2 ml acetonitrile:water (3:1 v/v) solution and infused with a syringe pump at flow rate of 30 μl/minute. The analyses were performed on a triple quadrupole instrument (model 310, Varian Inc./Agilent Technologies) with atmospheric pressure chemical ionization (APCI) source. The data were scanned in the range of 360–450 *m/z*. Nitrogen was used as nebulizer (275.8 KPa) and drying gas (68.9 KPa). Vaporization temperature was set at 300°C with the following conditions: capillary voltage set at 56 V, housing temperature set at 50°C, corona at 1μA and shield at 600 V. Sterol masses were retrieved from values of [M+H–H_2_O] and sterol abundance was assessed in non-saturated conditions. Data were acquired and analyzed with the Varian Workstation software version MS 6.9 and the amount of cholesterol and its precursors was assessed qualitatively comparing nonsilenced and ATP6V_0_d2-KD macrophages.

### Macrophage viability assays

Nonsilenced and ATP6V_0_d2-KD macrophages cultivated in 96-well plates were treated or not with different concentrations of ox-LDL for 48 hours. Next, samples were cultivated in a solution of 1 mg/ml 3-(4,5-dimethylthiazol-2-yl)-2,5-diphenyltetrazolium bromide (MTT, Sigma-Aldrich Inc.) for 2 hours in 37°C and 5% CO_2_. Macrophage supernatant chromogenic reaction was read at 540 nm in a micro ELISA reader (Multiskan MS–LabSystems, Finland). Cytotoxicity was assessed at cellular level by FACS using 1:1000 (v/v) of the viability dye eFluor780 (eBiosciences) following manufacturer instructions. For cell death positive control, macrophages were first fixed with 4% PFA for 15 minutes and then labeled with viability dye.

### Assessment of nitric oxide and cytokine production

Nonsilenced and ATP6V_0_d2-KD macrophages were cultivated in 24-well plates in complete medium stimulated or not with IFN-γ/LPS or ox-LDL for 48 hours. Cell culture supernatants were collected and stored at -80°C until analysis. Nitric oxide concentrations from 25 μl of supernatants were assessed as described [99] using the chemoluminescence reader Nitric Oxide Analyzer (NOA 208i –Sievers). To determine cytokine concentrations, supernatants were loaded with the Milliplex Map Mouse Cytokine/Chemokine Magnetic Bead Panel and Milliplex Map TGFβ1 Single Plex Magnetic Bead Kit (MCYTOMAG-70K and TGFBMAG-64K-01, Merck Milipore), following the manufacturer’s instructions. Samples were then analyzed by Luminex MAGPIX System 40–072 (Merck Millipore). The NO and cytokine concentrations were normalized according to the macrophage protein lysate concentration, as assessed using the Bradford method.

### Statistical analysis

The experiments were repeated independently at least twice using experimental replicates. The results were represented as the means with respective standard errors. Statistical tests were performed by SPSS software (IBM), considering normal (parametric tests) or nonnormal distributions (nonparametric tests), and significant differences were indicated by *p* values below 0.05. Data were normalized by nonsilenced or nontreated controls as indicated.

## Supporting information

S1 Fig Strategy for retrieving phagosomal pH measurements from FITC-tagged beads in GFP-expressing macrophages. **A.** Excitation and emission spectra of turboGFP (excitation maximum = 482 nm) and FITC (ex. max = 495 nm). When excited by a 496 nm laser, FITC emission yield is 2.7 higher than turboGFP’s using the same laser and the same emission range of 520–537 nm. Excitation spectra are shown as lines of GFP^ex^ and FITC^ex^, and emission spectra are shown as curves of GFP^em^ and FITC^em^. Spectra are shown as excitation and emission efficiencies relative to wavelengths (nm) as retrieved from www.fpbase.org/spectra/. **B.** FITC-tagged beads interacting with GFP-expressing macrophages. The differences in the excitation maximum of turboGFP and FITC using 496nm laser for excitation allowed us to adjust the voltage (gain) of photodetectors to threshold out most of turboGFP emission and some emission of FITC (first image, emission acquired using 500–520 nm detector). When emission detector was adjusted to collect fluorescence from 520-537nm, a FITC-specific signal is obtained (second image). The third image shows the merged signal obtained from the two detector configurations, namely turboGFP+FITC and FITC only. **C.** Histogram distribution showing the frequencies of pH measurements per FITC-tagged bead in nonsilenced or ATP6V_0_d2-KD macrophages. A pH>6.5 is detected only in 5% of the beads recorded. **D.** Live DIC and fluorescence images of FITC-tagged beads internalized by GFP-expressing macrophages after thoroughly washing out non-adhered and non-internalized beads. FITC-tagged beads remain associated with >50% of macrophages and the large majority of these beads display the characteristic decrease of FITC intensity related to acidic pH of phagolysosomes (pink arrowheads).
(TIF)

S2 Fig Activity of lysosomal enzymes in ATP6V_0_d2-KD macrophages. **A.** Confocal microscopy images on the right show double labeling of CTSD (green) and LAMP-1 (red) in nonsilenced or ATP6V_0_d2-KD macrophages. CTSD/LAMP-1 colocalization was performed from these confocal images and is represented as Pearson’s correlation coefficients, showing a stronger association of CTSD with lysosomes in ATP6V_0_d2-KD compared with nonsilenced macrophages. **B.** Western blotting for CTSD expressed by nonsilenced or ATP6V_0_d2-KD macrophages, indicating the absence of mature forms (30kDa band) in knock-down macrophages. LAMP-1 (110kDa band) was used to control the loaded sample concentration. **C.** Enzymatic activity of the lysosomal enzymes lysosomal acid lipase (LAL), α-galactosidase (α-Gal), and β-glucocerebrosidase (GCase) assessed in nonsilenced or ATP6V_0_d2-KD macrophages. The data were normalized by the maximum value obtained in nonsilenced macrophage per enzyme tested. The asterisks indicate statistical significance (p<0.05) between nonsilenced and ATP6V_0_d2-KD measurements. The results are representative of 3 independent experiments. **D.** Enzymatic activity of α-Gal and GCase assessed in nonsilenced or ATP6V_0_d2-KD macrophages infected or not by *L*. *amazonensis* for 72 hours, treated or not with 50 μg/ml ox-LDL for 48 hours during intracellular infection.
(TIF)

S3 Fig Strategy for IFN-γ/LPS classical activation or ox-LDL-mediated cholesterol replenishment for infected macrophages. **A.** Classical inflammatory activation was performed by treating non-infected macrophages with IFN-γ/LPS for 24 hours prior to 48 or 72 hours of intracellular infection according to the experiment. **B.** Cholesterol replenishment and PV volume restoration in the ATP6V_0_d2-KD model were performed by first infecting macrophages for 24 hours and then incubating infected macrophages in complete medium containing ox-LDL for the next 48 hours. In this strategy, the period of intracellular infection is 72 hours, comprising 48 hours of ox-LDL-mediated cholesterol replenishment.
(TIF)

S4 Fig The impairment of PV volumetric expansion in ATP6V_0_d2-KD macrophages is not associated with cell sphericity or acquisition of the late-endosomal SNARE VAMP8. **A.** Live imaging microscopic fields presenting the population of nonsilenced or ATP6V_0_d2-KD macrophages (green) infected by *L*. *amazonensis* (red) assessed by three-dimensional projections in *xy*, *xz* and *yz* coordinates, in addition to images presented in Fig 3C. Images reinforce the participation of ATP6V_0_d2 in controlling *L*. *amazonensis* PV expansion and indicate that macrophage models present similar morphology. Nuclei staining by Hoechst dye. Bar = 20 μm. **B.** Cell sphericity retrieved from infected nonsilenced and ATP6V_0_d2-KD macrophages during 36 hours of multidimensional live image acquisition. **C.** Scheme comparing area-based and volume-based strategies for morphometric assessment of PV sizes. Theoretically, although isosurfaces V_1_ and V_2_ display the same volumes, flat cells will have PVs with a spherical cap morphology and round cells will form PVs with a prolate spheroid morphology, providing different measures for PV areas A_1_ and A_2_. The focal plane chosen for PV area measurement in flat cells will be closer to the base of the spherical cap PV while focal planes chosen for the same measurement in round cells will be at the hemisphere. This approach will provide different area values for PVs displaying the same volume, and is only valid for cells presenting the same morphology. For this reason, PV volumetric assessment in three-dimensional or multidimensional images is a more accurate and reliable method for PV size assessment and comparison. This strategy for PV volume measurements was applied to ATP6V_0_d2-KD macrophages infected for up to 72 hours, activated or not with IFN-γ/LPS or treated or not with ox-LDL (graph on the lower panel), demonstrating that ox-LDL treatment efficiently restores PV dimensions to the sizes retrieved in nonsilenced non-activated macrophages. **D.** Acquisition of VAMP8 SNARE by zymosan phagolysosomes and *L*. *amazonensis* PVs after 48 hours of particle or parasite interaction with nonsilenced or ATP6V_0_d2-KD macrophages. Upper panel shows immunofluorescence images of VAMP8 associated with these phagolysosomes (arrowhead); VAMP8 immunostaining in red, nuclei in blue stained by DAPI. Lower panel shows the percentage of VAMP8^+^ zymosan phagolysosomes and *L*. *amazonensis* PVs presented in nonsilenced or ATP6V_0_d2-KD macrophages activated or not with IFN-γ/LPS. The asterisks indicate statistical significance (p<0.05). ns = nonsignificant. Representative of 2 independent experiments.
(TIF)

S5 Fig ATP6V_0_d2-KD macrophages present decreased levels of intracellular cholesterol, which are efficiently replenished by ox-LDL treatment. **A**. Relative abundance of cholesterol, squalene and lanosterol observed in nonsilenced and ATP6V_0_d2-KD macrophages as assessed by mass spectrometry using Atmospheric Pressure Chemical Ionization (APCI) in positive mode. A decrease in abundance of the m/z 369 ion (corresponding to cholesterol) is more pronounced than the subtle differences in the ions 393 and 409 corresponding to cholesterol precursors squalene and lanosterol respectively. Representative data of 2 independent experiments. **B.** Intracellular cholesterol levels (assessed by ELISA) and cell viability (assessed by MTT assay) of nonsilenced and ATP6V_0_d2-KD macrophages treated or not with different concentrations of methyl-β-cyclodextrin/cholesterol complexes (1.25 and 2.5 mM), nonmodified or oxidized LDL (25, 50 and 100 μg/ml) for 3 hours. The third lower graph shows the macrophage viability assessed by MTT after 48 hours of LDL or ox-LDL treatment using different concentrations. Treatment with 50 and 100 μg/ml of ox-LDL is the more efficient strategy to replenish cholesterol, which increased its intracellular levels without interfering in macrophage viability. **C.** Cell viability assessed at the cellular level by flow cytometry using viability dyes. Results are presented as the histogram of viability dye fluorescence intensities per condition, evaluating infected or non-infected nonsilenced and ATP6V_0_d2-KD macrophages, treated or not with 50 μg/ml of ox-LDL for 48 hours. Cell death positive controls (dead cell control) are provided by paraformaldehyde-fixed macrophages stained with the dye.
(TIF)

S6 Fig ATP6V_0_d2 does not participate in either the ubiquitous isoform ATP6V_0_d1, SREBP2 and LYST/Beige expression or in the production of nitric oxide or inflammatory cytokines in response to ox-LDL. **A-B.** ATP6V_0_d2 and ATP6V_0_d1 (A) or SREBP2 and LYST/Beige (B) mRNA expression in nonsilenced or ATP6V_0_d2-KD macrophages infected or not by *L*. *amazonensis* for 72 hours, treated or not with 50 μg/ml ox-LDL for 48 hours during intracellular infection. The results are representative of 2 independent experiments. **C.** Nitric oxide (NO, μM/mg of cell lysate) and cytokine production (μg/mg of cell lysate) detected in culture supernatants of nonsilenced or ATP6V_0_d2-KD macrophages activated or not with IFN-γ/LPS and treated or not with 50 μg/ml ox-LDL. IFN-γ/LPS significantly induced ATP6V_0_d2-independent production of NO and IFN-γ, IL-6 and TNF-α inflammatory cytokines compared with nontreated cells (p<0.05). Ox-LDL treatment is inefficient at triggering NO or inflammatory cytokine production (p>0.05 in comparison with ox-LDL-treated and nontreated macrophages).
(TIF)

S7 Fig Detailed information regarding gene expression analysis in accordance with MIQE guidelines. **A.** Table showing the efficiency of all the primers used in the study with the values of slope, R^2^ and percentage of efficiency. The parameter between the curves of target and endogenous genes of a standard curve is used to calculate the amplification efficiency of the reaction, according to the equation: E = [10^(-1 / slope)^– 1] x 100. **B.** Standard curves obtained by linear regression of the Ct amplification (cycle threshold) value on the log of the initial cDNA amount (quantity). An angular coefficient of the standard curve of -3.32 indicates a reaction with 100% efficiency. **C.** Melt curves of each gene analyzed in qPCR reactions demonstrating the specificity of the reaction. **D.** ATP6V_0_d2 mRNA expression as assessed by normalization using two different endogenous genes, β-actin (upper) and HPRT (lower graph), showing that the profile of the results is similar using both endogenous genes.
(TIF)

S1 Movie Live imaging of IFN-γ/LPS-activated nonsilenced (left) or ATP6V_0_d2-KD macrophages (right) hosting *L*. *amazonensis*. GFP-expressing macrophages in green and DsRed2-expressing parasites in red. PV volumetric expansion is observed in nonsilenced macrophages; smaller PVs, tight-fitting PVs and PV fission are observed in ATP6V_0_d2-KD macrophages. Time of image acquisition is expressed as days:hours:minutes:seconds:miliseconds (dh:mm:ss:sss). Bar = 30 μm.
(MOV)

S2 Movie Time-lapse imaging in differential interference contrast of infected nonsilenced (upper) or ATP6V_0_d2-KD macrophages (lower) treated with 50 μg/ml ox-LDL. Image acquisition started 24 hours post-infection and 15 minutes after ox-LDL addition. In the upper video, parasites multiply in large PVs in nonsilenced macrophages in the presence of ox-LDL; in the lower video, amastigote morphology indicates destruction of parasites within PVs in which volume was restored in ox-LDL-treated ATP6V_0_d2-KD macrophages. Time is represented as hours:minutes (h:mm). Bar = 10 μm.
(MOV)
