# Supplementary figures and images for: ATP6V0d2 controls Leishmania parasitophorous vacuole biogenesis via cholesterol homeostasis
Source: PLoS Pathog. 2019 Jun 14;15(6):e1007834. doi: 10.1371/journal.ppat.1007834 (PMC6594656; doi:10.1371/journal.ppat.1007834)

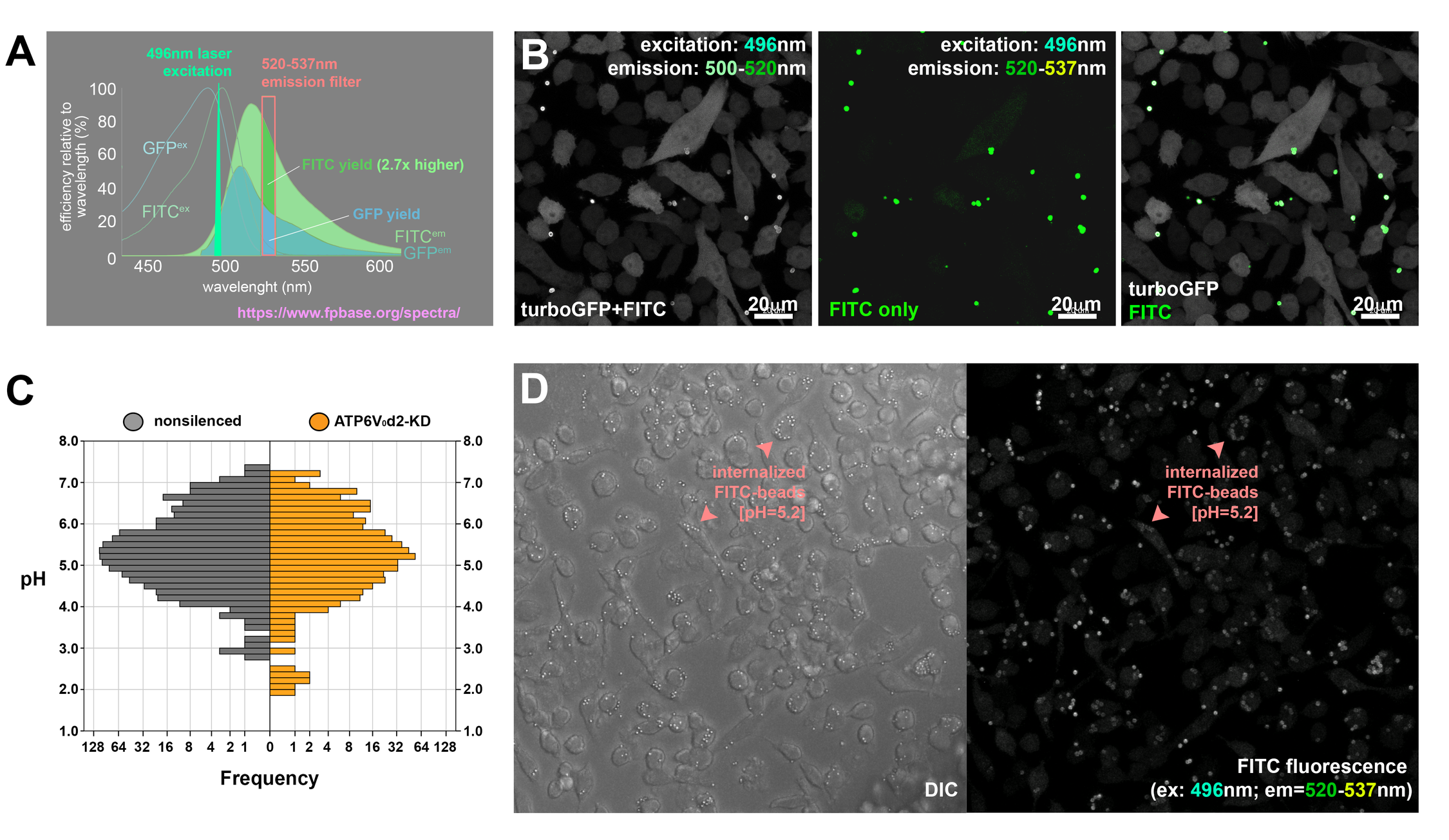

Supplement: S1 Fig — A. Excitation and emission spectra of turboGFP (excitation maximum = 482 nm) and FITC (ex. max = 495 nm). When excited by a 496 nm laser, FITC emission yield is 2.7 higher than turboGFP’s using the same laser and the same emission range of 520–537 nm. Excitation spectra are shown as lines of GFPex and FITCex, and emission spectra are shown as curves of GFPem and FITCem. Spectra are shown as excitation and emission efficiencies relative to wavelengths (nm) as retrieved from www.fpbase.org/spectra/. B. FITC-tagged beads interacting with GFP-expressing macrophages. The differences in the excitation maximum of turboGFP and FITC using 496nm laser for excitation allowed us to adjust the voltage (gain) of photodetectors to threshold out most of turboGFP emission and some emission of FITC (first image, emission acquired using 500–520 nm detector). When emission detector was adjusted to collect fluorescence from 520-537nm, a FITC-specific signal is obtained (second image). The third image shows the merged signal obtained from the two detector configurations, namely turboGFP+FITC and FITC only. C. Histogram distribution showing the frequencies of pH measurements per FITC-tagged bead in nonsilenced or ATP6V0d2-KD macrophages. A pH>6.5 is detected only in 5% of the beads recorded. D. Live DIC and fluorescence images of FITC-tagged beads internalized by GFP-expressing macrophages after thoroughly washing out non-adhered and non-internalized beads. FITC-tagged beads remain associated with >50% of macrophages and the large majority of these beads display the characteristic decrease of FITC intensity related to acidic pH of phagolysosomes (pink arrowheads). (TIF) [file ppat.1007834.s001.tif]

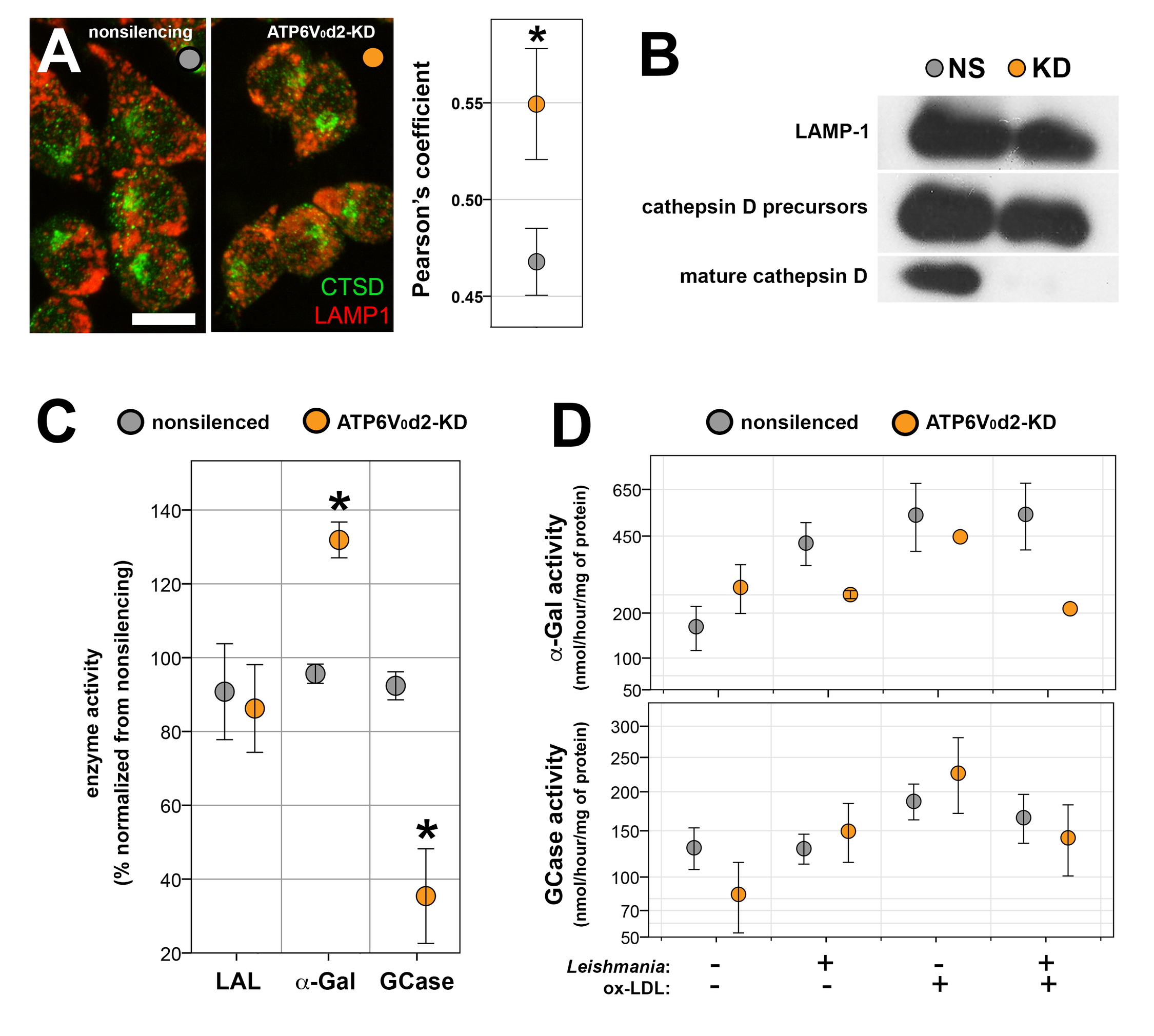

Supplement: S2 Fig — A. Confocal microscopy images on the right show double labeling of CTSD (green) and LAMP-1 (red) in nonsilenced or ATP6V0d2-KD macrophages. CTSD/LAMP-1 colocalization was performed from these confocal images and is represented as Pearson’s correlation coefficients, showing a stronger association of CTSD with lysosomes in ATP6V0d2-KD compared with nonsilenced macrophages. B. Western blotting for CTSD expressed by nonsilenced or ATP6V0d2-KD macrophages, indicating the absence of mature forms (30kDa band) in knock-down macrophages. LAMP-1 (110kDa band) was used to control the loaded sample concentration. C. Enzymatic activity of the lysosomal enzymes lysosomal acid lipase (LAL), α-galactosidase (α-Gal), and β-glucocerebrosidase (GCase) assessed in nonsilenced or ATP6V0d2-KD macrophages. The data were normalized by the maximum value obtained in nonsilenced macrophage per enzyme tested. The asterisks indicate statistical significance (p<0.05) between nonsilenced and ATP6V0d2-KD measurements. The results are representative of 3 independent experiments. D. Enzymatic activity of α-Gal and GCase assessed in nonsilenced or ATP6V0d2-KD macrophages infected or not by L. amazonensis for 72 hours, treated or not with 50 μg/ml ox-LDL for 48 hours during intracellular infection. (TIF) [file ppat.1007834.s002.tif]

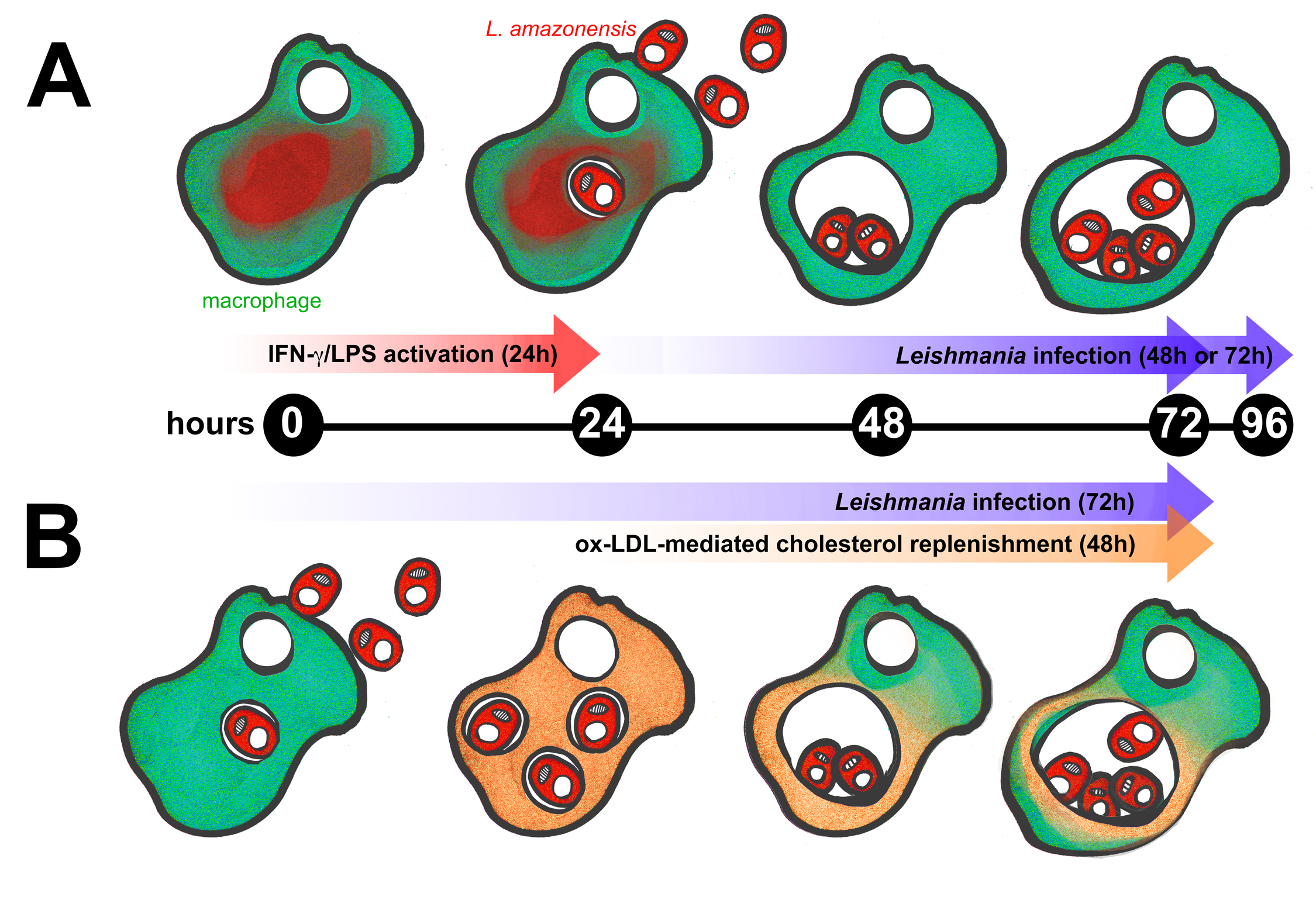

Supplement: S3 Fig — A. Classical inflammatory activation was performed by treating non-infected macrophages with IFN-γ/LPS for 24 hours prior to 48 or 72 hours of intracellular infection according to the experiment. B. Cholesterol replenishment and PV volume restoration in the ATP6V0d2-KD model were performed by first infecting macrophages for 24 hours and then incubating infected macrophages in complete medium containing ox-LDL for the next 48 hours. In this strategy, the period of intracellular infection is 72 hours, comprising 48 hours of ox-LDL-mediated cholesterol replenishment. (TIF) [file ppat.1007834.s003.tif]

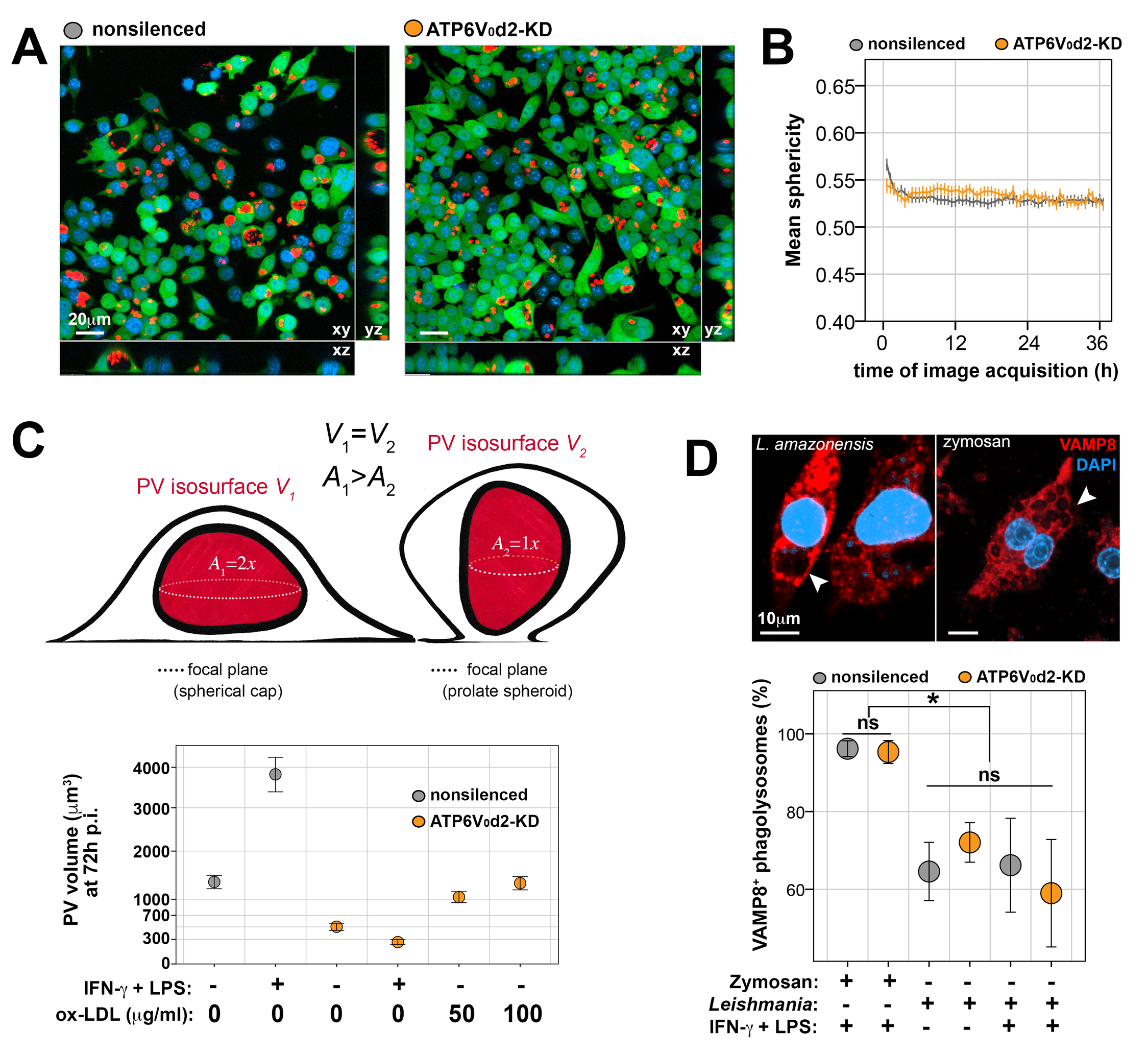

Supplement: S4 Fig — A. Live imaging microscopic fields presenting the population of nonsilenced or ATP6V0d2-KD macrophages (green) infected by L. amazonensis (red) assessed by three-dimensional projections in xy, xz and yz coordinates, in addition to images presented in Fig 3C. Images reinforce the participation of ATP6V0d2 in controlling L. amazonensis PV expansion and indicate that macrophage models present similar morphology. Nuclei staining by Hoechst dye. Bar = 20 μm. B. Cell sphericity retrieved from infected nonsilenced and ATP6V0d2-KD macrophages during 36 hours of multidimensional live image acquisition. C. Scheme comparing area-based and volume-based strategies for morphometric assessment of PV sizes. Theoretically, although isosurfaces V1 and V2 display the same volumes, flat cells will have PVs with a spherical cap morphology and round cells will form PVs with a prolate spheroid morphology, providing different measures for PV areas A1 and A2. The focal plane chosen for PV area measurement in flat cells will be closer to the base of the spherical cap PV while focal planes chosen for the same measurement in round cells will be at the hemisphere. This approach will provide different area values for PVs displaying the same volume, and is only valid for cells presenting the same morphology. For this reason, PV volumetric assessment in three-dimensional or multidimensional images is a more accurate and reliable method for PV size assessment and comparison. This strategy for PV volume measurements was applied to ATP6V0d2-KD macrophages infected for up to 72 hours, activated or not with IFN-γ/LPS or treated or not with ox-LDL (graph on the lower panel), demonstrating that ox-LDL treatment efficiently restores PV dimensions to the sizes retrieved in nonsilenced non-activated macrophages. D. Acquisition of VAMP8 SNARE by zymosan phagolysosomes and L. amazonensis PVs after 48 hours of particle or parasite interaction with nonsilenced or ATP6V0d2-KD macrophages. Upper panel shows immun [file ppat.1007834.s004.tif]

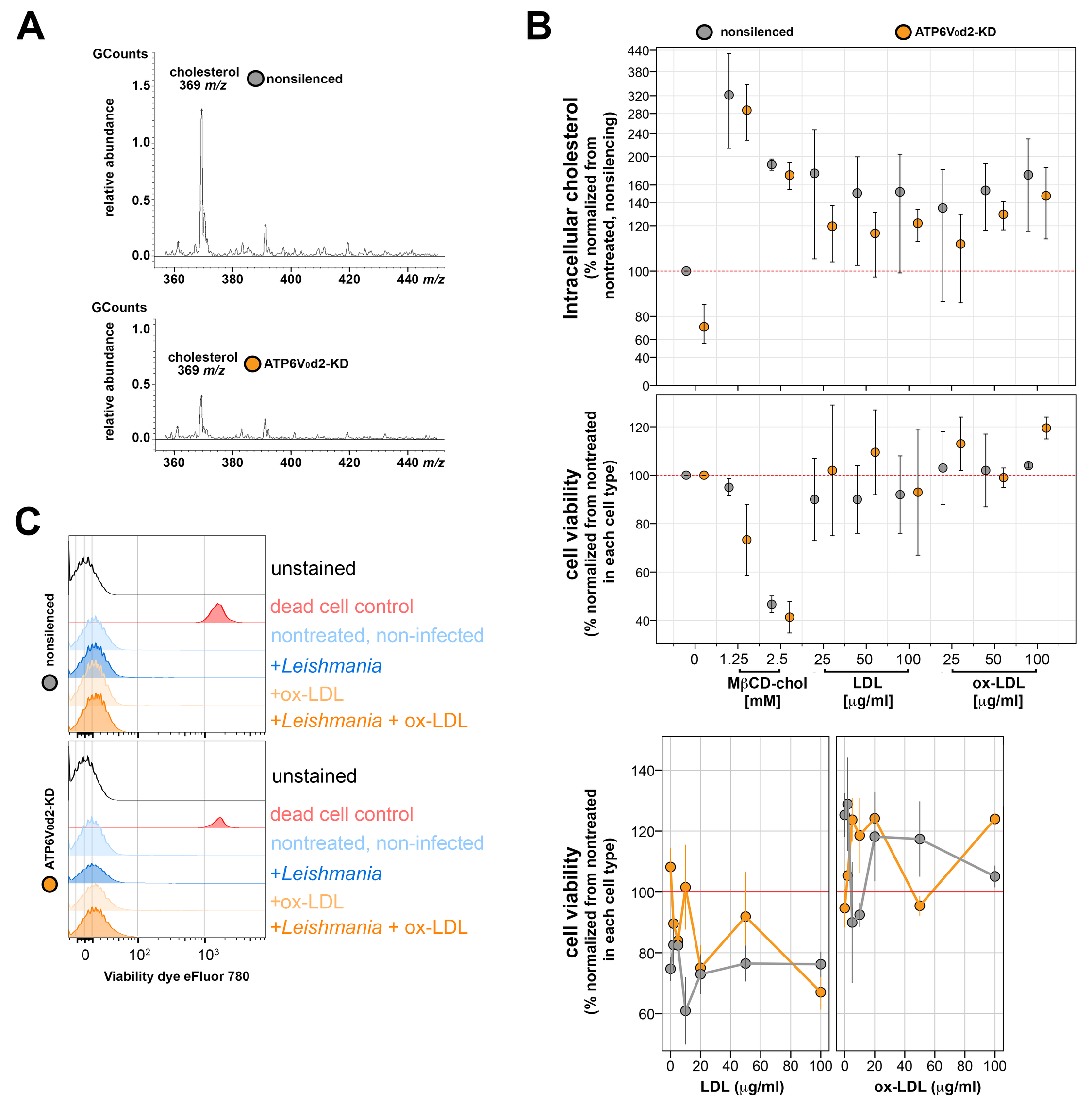

Supplement: S5 Fig — A. Relative abundance of cholesterol, squalene and lanosterol observed in nonsilenced and ATP6V0d2-KD macrophages as assessed by mass spectrometry using Atmospheric Pressure Chemical Ionization (APCI) in positive mode. A decrease in abundance of the m/z 369 ion (corresponding to cholesterol) is more pronounced than the subtle differences in the ions 393 and 409 corresponding to cholesterol precursors squalene and lanosterol respectively. Representative data of 2 independent experiments. B. Intracellular cholesterol levels (assessed by ELISA) and cell viability (assessed by MTT assay) of nonsilenced and ATP6V0d2-KD macrophages treated or not with different concentrations of methyl-β-cyclodextrin/cholesterol complexes (1.25 and 2.5 mM), nonmodified or oxidized LDL (25, 50 and 100 μg/ml) for 3 hours. The third lower graph shows the macrophage viability assessed by MTT after 48 hours of LDL or ox-LDL treatment using different concentrations. Treatment with 50 and 100 μg/ml of ox-LDL is the more efficient strategy to replenish cholesterol, which increased its intracellular levels without interfering in macrophage viability. C. Cell viability assessed at the cellular level by flow cytometry using viability dyes. Results are presented as the histogram of viability dye fluorescence intensities per condition, evaluating infected or non-infected nonsilenced and ATP6V0d2-KD macrophages, treated or not with 50 μg/ml of ox-LDL for 48 hours. Cell death positive controls (dead cell control) are provided by paraformaldehyde-fixed macrophages stained with the dye. (TIF) [file ppat.1007834.s005.tif]

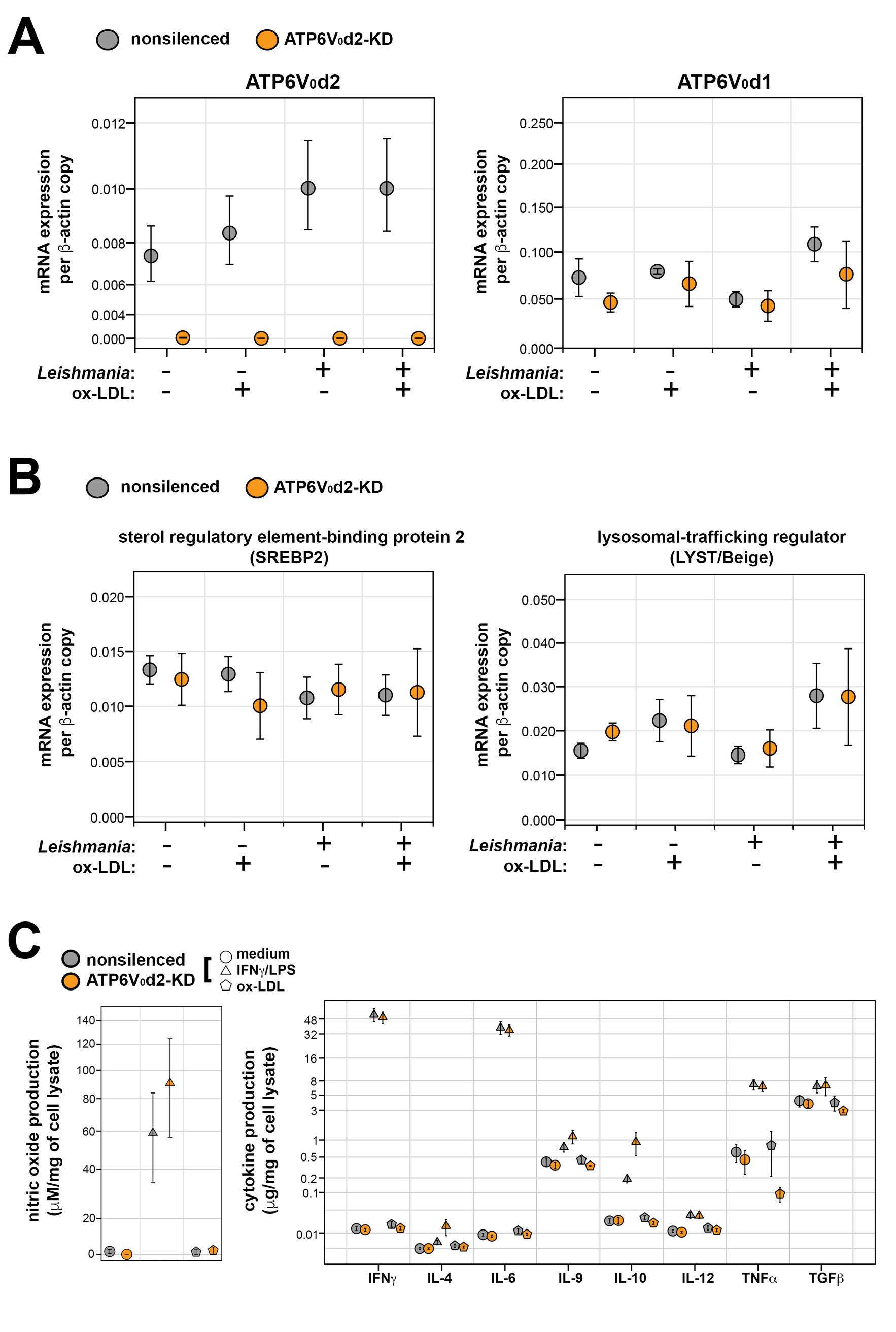

Supplement: S6 Fig — A-B. ATP6V0d2 and ATP6V0d1 (A) or SREBP2 and LYST/Beige (B) mRNA expression in nonsilenced or ATP6V0d2-KD macrophages infected or not by L. amazonensis for 72 hours, treated or not with 50 μg/ml ox-LDL for 48 hours during intracellular infection. The results are representative of 2 independent experiments. C. Nitric oxide (NO, μM/mg of cell lysate) and cytokine production (μg/mg of cell lysate) detected in culture supernatants of nonsilenced or ATP6V0d2-KD macrophages activated or not with IFN-γ/LPS and treated or not with 50 μg/ml ox-LDL. IFN-γ/LPS significantly induced ATP6V0d2-independent production of NO and IFN-γ, IL-6 and TNF-α inflammatory cytokines compared with nontreated cells (p<0.05). Ox-LDL treatment is inefficient at triggering NO or inflammatory cytokine production (p>0.05 in comparison with ox-LDL-treated and nontreated macrophages). (TIF) [file ppat.1007834.s006.tif]

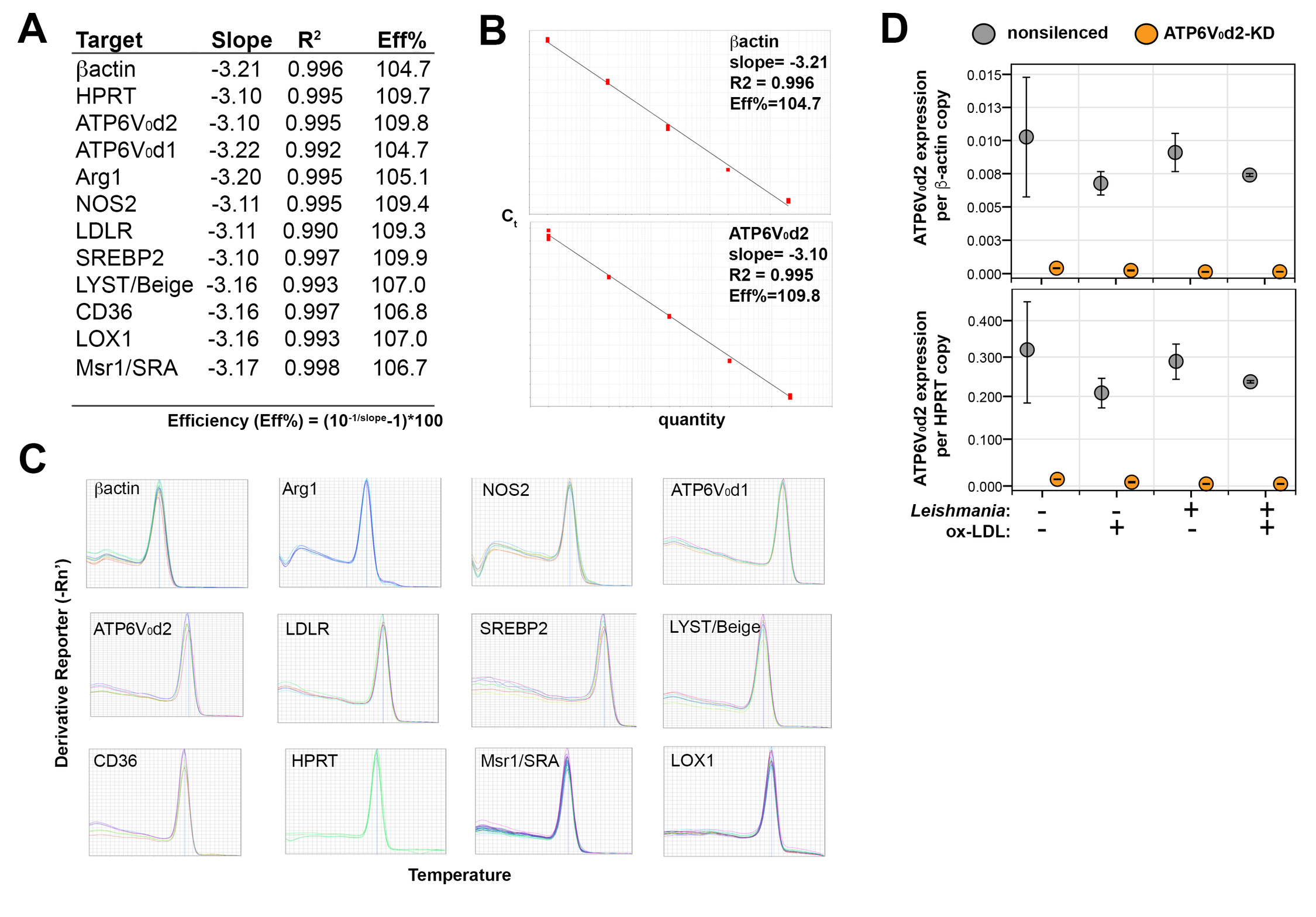

Supplement: S7 Fig — A. Table showing the efficiency of all the primers used in the study with the values of slope, R2 and percentage of efficiency. The parameter between the curves of target and endogenous genes of a standard curve is used to calculate the amplification efficiency of the reaction, according to the equation: E = [10(-1 / slope)– 1] x 100. B. Standard curves obtained by linear regression of the Ct amplification (cycle threshold) value on the log of the initial cDNA amount (quantity). An angular coefficient of the standard curve of -3.32 indicates a reaction with 100% efficiency. C. Melt curves of each gene analyzed in qPCR reactions demonstrating the specificity of the reaction. D. ATP6V0d2 mRNA expression as assessed by normalization using two different endogenous genes, β-actin (upper) and HPRT (lower graph), showing that the profile of the results is similar using both endogenous genes. (TIF) [file ppat.1007834.s007.tif]
